# Decreased Cross-Domain Mutual Information in Schizophrenia From Dynamic Connectivity States

**DOI:** 10.3389/fnins.2019.00873

**Published:** 2019-08-22

**Authors:** Mustafa S. Salman, Victor M. Vergara, Eswar Damaraju, Vince D. Calhoun

**Affiliations:** ^1^School of Electrical and Computer Engineering, Georgia Institute of Technology, Atlanta, GA, United States; ^2^Tri-Institutional Center for Translational Research in Neuroimaging and Data Science (TReNDS), Georgia State University, Georgia Institute of Technology and Emory University, Atlanta, GA, United States

**Keywords:** fMRI, functional network connectivity, functional domain, ICA, schizophrenia, information theory

## Abstract

The study of dynamic functional network connectivity (dFNC) has been important to understand the healthy and diseased brain. Recent developments model groups of functionally related brain structures (defined as functional domains) as entities that can send and receive information. A domain analysis starts by detecting a finite set of connectivity patterns known as domain states within each functional domain. Dynamic functional domain connectivity (DFDC) is a novel information theoretic framework for studying the temporal sequence of the domain states and the amount of information shared among domains. In this setting, the information flow among functional domains can be compared to the flow of bits among entities in a digital network. Schizophrenia is a chronic psychiatric disorder which is associated with how the brain processes information. Here, we employed the DFDC framework to analyze a dataset containing resting-state fMRI scans from 163 healthy controls (HCs) and 151 schizophrenia patients (SZs). As in other information theory methods, this study measured domain state probabilities, entropy within each DFDC and the cross-domain mutual information (CDMI) between pairs of DFDC. Results indicate that SZs show significantly higher (transformed) entropy than HCs in subcortical (SC)-SC; default mode network (DMN)-visual (VIS) and frontoparietal (FRN)-VIS DFDCs. SZs also show lower (transformed) CDMI between SC-VIS vs. SC-sensorimotor (SM), attention (ATTN)-VIS vs. ATTN-SM and ATTN-SM vs. ATTN-ATTN DFDC pairs after correcting for multiple comparisons. These results imply that different DFDC pairs function in a more independent manner in SZs compared to HCs. Our findings present evidence of higher uncertainty and randomness in SZ brain function.

## Introduction

Schizophrenia is a chronic psychiatric disorder whose mechanism is not well understood yet. Its pathological and genetic background is complex and has not provided a clear understanding of the cause of the disorder. As a result, interest is growing in the functional neuroimaging of schizophrenia to provide additional clues about how this disorder impacts brain function (Kahn et al., 2015). The symptoms of schizophrenia can be broadly divided into positive, negative and cognitive categories. For instance, positive symptoms, characterized by abnormal salience processing and hallucinations, have shown links to abnormal functional activation in midbrain, speech and auditory cortices in SZs (Murray et al., 2008; Sommer et al., 2008). Negative symptoms related to reward processing and social cognition have shown association with reduced activation in amygdala in SZs (Aleman and Kahn, 2005; Juckel et al., 2006). Broad impairment of cognitive function, such as working and episodic memory, manifest as abnormal activation in dorsolateral prefrontal cortex in SZs (Brunet-Gouet and Decety, 2006; Fusar-Poli et al., 2013). Recent results suggest that these regional alterations are best understood as abnormalities in functional connectivity. For example, dorsolateral prefrontal cortex connectivity is altered in SZs and individuals at risk (Wolf et al., 2009; Esslinger et al., 2013). Methods from network topology has shown that small-world properties may be altered in SZs (Bassett et al., 2008; van den Heuvel et al., 2013). Schizophrenia is therefore associated with subtle changes in neural cell population and cell to cell communication and the study of brain functional connectivity is critical in understanding its causes.

Functional magnetic resonance imaging is a non-invasive imaging technique which can be used to study normal brain function in healthy individuals and disrupted brain function in patients with brain disorders. It captures signals from brain regions using a BOLD contrast in response to tasks or at rest. Resting-state fMRI reveals that even in the absence of a task, anatomically separate brain regions show large-scale neuronal activity which are functionally synchronized to one another (Biswal et al., 1995; Greicius et al., 2003). FNC refers to the temporal coherence of low-frequency BOLD activity between separate networks of neurons. The temporal coherence also shows variation with time thus characterizing a dFNC signal (Allen et al., 2014). Two of the most widely used methods for the analysis of FNC are seed-based approaches (Biswal et al., 1995) and data-driven approaches. Among the data driven approaches, ICA is in widespread use (Calhoun et al., 2009). The spatial ICA approach expresses the temporal fMRI data as a linear combination of spatially (statistically) independent sources known as functional networks (hereafter referred to as simply “networks”) (Erhardt et al., 2011a). Unlike a seed-based approach, ICA requires no prior information about the pattern of the networks or their TCs. In this work, we perform ICA analysis of multi-subject fMRI data using the group ICA approach (Calhoun et al., 2001; Calhoun and Adali, 2012). This approach allows for estimation of the spatial networks common across subjects, and the associated TCs. These TCs can then be used to study dynamic brain function in different populations.

A growing number of studies of dynamic brain function using data-driven analysis of fMRI have shown links to the genetic risks, brain biology and clinical state of schizophrenia. Common approaches for studying dFNC of the human brain identifies replicable patterns of correlation among brain regions, known as brain states (Allen et al., 2014; Barttfeld et al., 2015; Leonardi and Van De Ville, 2015; Abrol et al., 2016; Chen et al., 2017). It has been found that SZs tend to linger in a state of weak connectivity at rest (Damaraju et al., 2014; Du et al., 2016; Lottman et al., 2017; Rabany et al., 2018; Sanfratello et al., 2019). Similar findings have been reported in patients with bipolar disorder and mild cognitive impairment (Rashid et al., 2016; Zhi et al., 2018). SZs also demonstrate reduced connectivity dynamism in the higher dimensional meta-state space compared to the HCs (Miller et al., 2016b). Another prior study indicated that the combined auditory-visual-sensorimotor network in HCs show increased sensitivity to connectivity in the other functional domains (groups of functionally related brain networks) in terms of measures derived by taking the transition probabilities of the inter-domain and intra-domain correlation patterns into account (Miller et al., 2016a). Group ICA methods have also identified potential biomarkers for schizophrenia, bipolar disorder and schizoaffective disorder (Du et al., 2015) and links with reduced brain volume and dFNC in schizophrenia (Abrol et al., 2017). These studies have demonstrated widespread disruption of interaction within the brain in schizophrenia and other mental disorders by employing whole-brain dFNC analysis.

Recently, Vergara et al. proposed an information theoretic framework to study communication between brain functional domains (Vergara and Calhoun, 2017; Vergara et al., 2017). Functional domains are groups of anatomically and functionally coherent networks. This approach allows for investigation of the flow of information between the functional domains by assigning binary digit values, or bits, to DFDC properties of the brain and estimating the quantity of bits required for this communication in terms of entropy and MI. The human brain is a large and complicated system and information theory can be a very useful statistical tool for studying such a system. Information theory was first described in Shannon’s seminal paper “A Mathematical Theory of Communication” (Shannon, 1948) and since then has been applied to diverse fields in science because of its significance and flexibility. The word “information” may convey different meaning in different context. In information theory, a random variable *X* provides (mutual) information about another random variable *Y* when the knowledge about *X* reduces the average uncertainty about *Y* (Cover and Thomas, 2006). MI is non-negative and symmetric, i.e., *X* says as much about *Y* as says about *X*. The average uncertainty in a random variable is known as entropy. These basic concepts of information theory may enable us to assign relative measures to how information is stored and transferred within the human brain. Indeed, much of the application of information theory in neuroscience is on neural information flow, which concerns the transmission and constraints of information flowing through the nervous system (Borst and Theunissen, 1999; Dimitrov et al., 2011). However, apart from the study of neural information flow, there is little application of information theory in the study of healthy and diseased brain function in the existing literature.

In this work, we use the information theoretic framework of DFDC to examine group differences between HCs and SZs. We first use the spatial group ICA technique to estimate spatially ICs and associated TCs from the fMRI scans of 163 HCs and 151 SZs. Then we use the TCs to estimate the whole-brain dFNC, from which we extract the DFDC states. We study various information theoretic properties of these time-varying DFDC states, such as state probabilities, entropy and CDMI. Finally, we conduct some statistical analysis on these properties to find links to schizophrenia.

## Materials and Methods

### Data

The data used in this study were collected under the FBIRN phase-III study at the following sites across the United States: University of California, Irvine (UCI), University of California, Los Angeles (UCLA); The University of New Mexico (NM), The University of Iowa (IA), University of Minnesota (MN), Duke University/University of North Carolina, University of California, San Diego (UCSD; healthy subjects only), and the University of California San Francisco (UCSF). Informed consent was obtained from the participants according to the guidelines set by the Internal Review Boards at each site. Resting-state fMRI data were originally collected from 186 HCs and 176 SZs. The SZ subjects were diagnosed using Structured Clinical Interview for DSM-IV-TR Axis I Disorders (First et al., 2002). The SZ subjects were excluded if they had a history of major medical illness and tardive dyskinesia or significant extrapyramidal symptoms or significant changes in psychotropic medications in the previous 2 months before the scan. Any healthy subject who had a first degree relative with a psychotic illness diagnosis or history of major neurological or psychiatric medical illness was also excluded. Subjects were also excluded if they did not have any of the following: sufficient eyesight to see visual displays, normal hearing levels, fluency in English and ability to perform the study tasks, IQ greater than 75 or if they had previous head injury or prolonged unconsciousness, substance or alcohol dependence, migraine treatments or MRI contradictions. Prior to participating in scanning procedures, all subjects received extensive diagnostic evaluations by experienced raters. All patients received the following symptom ratings: Scales for the Assessment of Positive (SAPS) and Negative Symptoms (SANS) and a modified Positive and Negative Symptom Scale (PANSS). The SZs were age and gender-matched. Imaging data for six of the seven sites were collected on a 3T Siemens Tim Trio System and on a 3T General Electric Discovery MR750 scanner at one site. 162 volumes of EPI, BOLD, fMRI data were collected from each participant using 3T scanners with the following imaging parameters: FOV = 220 mm × 220 mm (64 × 64 matrix), TR = 2 s, TE = 30 ms, FA = 770, 32 sequential ascending axial slices with thickness of 4 and 1 mm skip.

### Medication Information

Anti-psychotic data was available for 129 patients in the FBIRN phase-III dataset. We used their respective chlorpromazine (CPZ) dosage equivalents for the patients with available dose-level medication data, as specified by Andreasen et al. (2010).

### Preprocessing

We preprocessed the fMRI data using the SPM (Friston, 2007) and AFNI (Cox, 1996) toolboxes and scripts written in the MATLAB^TM^ software. At first, we measured the SFNR (Friedman et al., 2006) and maximum RMS translation using the INRIAlign toolbox in SPM (Freire et al., 2002) for all subjects. We excluded subjects with SFNR < 150 and RMS translation >4 mm, which left us with 314 subjects for analysis, including 163 HCs (mean age 36.9, 46 women) and 151 SZs (mean age 37.8, 37 women). Next, we performed slice-timing correction using the middle slice as reference, followed by despiking using AFNI’s 3dDespike algorithm to reduce the effect of outliers, normalization to the standard MNI space, resampling to 3 mm × 3 mm × 3 mm voxels, and smoothing to 6 mm FWHM kernel. Finally, we performed variance normalization on each voxel TC. More details about the data acquisition, preprocessing and quality control can be found in prior work (Damaraju et al., 2014; Keator et al., 2016).

### Group ICA

We performed group-level spatial ICA (Calhoun et al., 2001; Calhoun and Adali, 2012) to decompose the temporally concatenated fMRI data of all subjects into 100 spatially independent group-level components. This was achieved in two steps. At first, we performed a subject-level PCA with the number of PCs set at 120, followed by a group-level PCA on the reduced and concatenated data with the number of PCs set at 100. We chose a high number of PCs as it has been shown to stabilize the subsequent back-reconstruction process and produce refined ICs corresponding to known anatomical and functional segmentation (Allen et al., 2011; Erhardt et al., 2011b). Next, we performed ICA on the PCA-reduced data using the Infomax algorithm (Bell and Sejnowski, 1995) to estimate the group-level ICs. We repeated ICA 20 times and selected the most representative solution to ensure the stability of the IC estimation (Ma et al., 2011). We identified 100 most reliable components as the final group-level ICs.

After discarding the artifact-related ICs, we characterized 47 of the group-level ICs as RSN. An IC was identified as an RSN if its peak activation cluster was in the gray matter, there was minimal overlap with known vascular, susceptibility, ventricular and edge regions, and the mean power spectra of the IC showed higher low frequency spectral power (Allen et al., 2011). We grouped these networks into SC, auditory (AUD), SM, VIS, ATTN, frontal (FRN), DMN, and cerebellar (CB) functional domains based on their anatomical and presumed functional properties (Allen et al., 2014). Next, we estimated the subject-specific networks and their associated TCs based on the group-level ICs using the STR approach (Beckmann et al., 2009; Erhardt et al., 2011b). Finally, we post-processed the subject-level TCs of these networks by removing linear, quadratic and cubic trends, regressing out the head motion parameters and despiking using AFNI’s 3dDespike algorithm (Cox, 1996). Correlation among brain networks are known to be driven primarily by low frequency fluctuations in the BOLD fMRI data (Cordes et al., 2001). Therefore, we filtered the TCs using a 5th order Butterworth filter with a passband frequency of 0.01−0.15 Hz.

### dFNC Estimation

We proceeded to analyze dFNC for each subject using a sliding window method (Sakoğlu et al., 2010; Allen et al., 2014; Damaraju et al., 2014). We used a sliding window with size of 22 TR (44 s) and convolved it with a Gaussian of σ = 3 TR to obtain tapering along the edges. We used the sliding window in steps of 1 TR to estimate the dFNC among the TCs from a regularized inverse covariance matrix using a graphical LASSO framework (Friedman et al., 2008; Varoquaux et al., 2010). We transformed the dFNC values for each subject using the Fisher-Z transformation and residualized them with respect to the gender, age and site variables. The dFNC analysis was performed using the temporal dFNC toolbox included in the GIFT software (Calhoun, 2004). Prior work can be consulted to obtain more details on the group ICA analysis of the fMRI data and the dFNC estimation steps (Damaraju et al., 2014).

### Dynamic Functional Domain Connectivity

**FIGURE 1:**
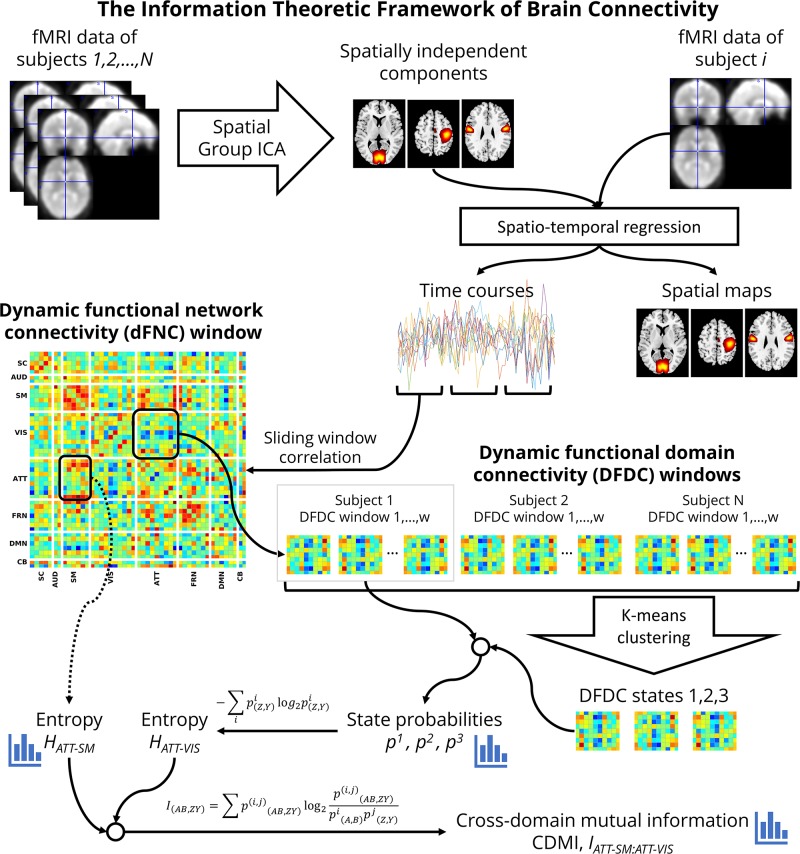
A flowchart of the information theoretic framework of DFDC.

Figure 1 shows a flowchart of the analysis undertaken to investigate schizophrenia using the information theoretic framework. At first, for each window, we used every connectivity between the networks belonging to each pair of domains *Z*[*t*] and *Y*[*t*] to determine the temporal DFDC matrix D_(_*_*Z,Y*_*_)_[*t*] (Vergara et al., 2017). Note that the FNC between two TCs is a scalar quantity, but the DFDC between two functional domains is not scalar unless both contain one network each. Furthermore, the FNC of a network with itself is 1 (one). In contrast, it is possible to have a multi-dimensional DFDC for a functional domain with itself if it contains more than two networks. In the two-network case, the DFDC is a scalar quantity. In our analysis, there were two such functional domains. The next step in our framework is to run a clustering algorithm on the DFDC. We chose not to apply clustering on the two DFDC with scalar quantities and excluded them from further analysis. We applied *K*-means clustering to the DFDC D_(_*_*Z,Y*_*_)_[*t*] across all windows and all subjects with 10 replicates. We set the number of clusters at *K* = 3 using the elbow criterion on the cluster validity index (Allen et al., 2014). Thus we identified the cluster centroids $C_{\left(Z,Y\right)}^{k}=\left[D_{\left(Z,Y\right)}^{1},D_{\left(Z,Y\right)}^{2},D_{\left(Z,Y\right)}^{3}\right]$ and a membership function $m_{\left(Z,Y\right)}\,\left[t\right]\in C_{\left(Z,Y\right)}^{k}$ corresponding to each windowed DFDC. The DFDC analysis was performed using custom scripts written in MATLAB^TM^.

Each cluster represents a dynamic state in DFDC. This method is analogous to the whole-brain dynamic state identification method (Allen et al., 2014), but estimates the dynamic states at the functional domain level. Therefore, as shown in Figure 1, DFDC is essentially a sub-matrix of the whole-brain dFNC matrix. The membership function expresses the closest cluster centroid based on an arbitrary distance measure. Here, we used correlation as the distance metric as it is a normalized metric showing more sensitivity to the DFDC patterns, although other possible choices such as *L*_1_, *L*_2_ norms will likely be highly similar (Damaraju et al., 2014).

### DFDC State Probabilities and Entropy

Next, we conceptualized the data within an information theoretic framework. In this framework, each of the DFDC states in the set *C*^*k*^_(_*_*Z,Y*_*_)_ is an element from an alphabet. The corresponding membership function *m*_(_*_*z,y*_*_)_[*t*] defines the frequency of occurrence of each dynamic state *D*^*i*(^*^*z,y*^*^)^ (alphabet element). Thus, we can estimate the probability, *p*^*i*(^*^*z,y*^*^)^, of a given state *D*^*i*(^*^*z,y*^*^)^ from the membership function (also known as the occupancy rate). The DFDC entropy is then computed as:

(1) $$H_{\left(Z,Y\right)}=-\sum_{i}p_{\left(Z,Y\right)}^{i}\,\log_{2}\left(p_{\left(Z,Y\right)}^{i}\right)$$

Entropy provides a measure of uncertainty. Higher entropy indicates that all possible states of a DFDC are nearly equally likely to be observed. Conversely, zero entropy implies maximum predictability of an outcome state.

#### dFNC Entropy

Just as for the DFDC states, we can also capture uncertainly at the whole-brain level by measuring entropy of the dFNC states. It is computed as:

(2) $$H_{\left(X\right)}=-\sum_{i}p_{\left(X\right)}^{i}\,\log_{2}\left(p_{\left(X\right)}^{i}\right)$$

Here, *p*^*i*^_(_*_*x*_*_)_ is the probability of a given dFNC state for a subject, where the states are estimated by running *K*-means clustering on the dFNC matrices across all subjects with *K* = 3. This is useful for interpreting the findings based on DFDC and dFNC.

### Cross-Domain Mutual Information

The other information theoretic measure we were interested in is the CDMI. CDMI is defined as:

(3) $$I_{\left(A\,B,Z\,Y\right)}=\sum p_{\left(A\,B,Z\,Y\right)}^{\left(i,j\right)}\,\log_{2}\frac{p_{\left(A\,B,Z\,Y\right)}^{\left(i,j\right)}}{p_{\left(A,B\right)}^{i}\,p_{\left(Z,Y\right)}^{j}}$$

where *A* and *B* are two other different domains than *Z* and *Y*. $p_{\left(A\,B,Z\,Y\right)}^{\left(i,j\right)}$ indicates the joint probability based on the temporal co-occurrence of the membership states *m*_(*Z*,*Y*)_[*t*],  *m*_(*A*,*B*)_[*t*] and is given by:

(4) $$p_{\left(A\,B,Z\,Y\right)}^{i}=p\left(m_{\left(Z,Y\right)}=D_{\left(Z,Y\right)}^{i},m_{\left(A,B\right)}=D_{\left(A,B\right)}^{i}\right)$$

(5) $$D_{\left(Z,Y\right)}^{i}\in C_{\left(Z,Y\right)}^{\left(k_{1}\right)},D_{\left(A,B\right)}^{i}\in C_{\left(A,B\right)}^{\left(k_{1}\right)}$$

It can be shown from Eq. 2 that*I*_(*AB*,*ZY*)_=*H*_(*Z*,*Y*)_−*H*_(*Z*,*Y*|*A*,*B*)_, where *H*_(_*_*Z,Y*_*_|_*_*A,B*_*_)_ is the conditional entropy of the DFDC *D*_(_*_*Z,Y*_*_)_ given *D*_(_*_*A*_*_,_*_*B*_*_)_. MI serves as a measure of dependence (or lack thereof) between two random variables. MI is symmetric and non-negative, and zero if and only if the two random variables are statistically independent. Note that if *Z*=*A* and *Y*=*B*, then *I*_*(AB,ZY)*_ simply indicates the entropy of *D*_*(Z,Y)*_, or *H*_*(Z,Y)*_.

### Statistical Analysis

We performed statistical analysis on various information theoretic measures, i.e., state probabilities, entropy and CDMI, with the intent of testing for group differences. We observed that the DFDC state probabilities had positively skewed distribution (see Supplementary Figures 2, 4, 6). In case of DFDC entropy, the distributions were negatively skewed (see Supplementary Figure 8). Note that the number of maximum possible dynamic states for each DFDC was set at 3, which translates to a maximum achievable entropy value of 1.585 bits/sample in the event of an equal occupancy rate (33.33%) for each state. We transformed the DFDC state probabilities and entropy data across all subjects in the following manner to obtain Gaussian distributions. At first the data was scaled between [−(1−*K*), (1−*K*)]. Here, *K* is a small number, introduced so that we could subsequently apply the Fisher-Z transformation on the scaled data without encountering discontinuity. Next, we used a one-sample Kolmogorov–Smirnov test to confirm that the transformed quantities came from a standard normal distribution and hence satisfied the assumptions of relevant statistical analyses. We fit a linear model separately to each transformed DFDC state probability and entropy with the subject diagnosis (HC-SZ) as the independent categorical variable and examined the group difference. We also looked at group difference in the whole-brain dFNC entropy using a similar test.

We observed that most of the CDMI were very low, indicating high statistical independence between the corresponding DFDC pairs. We used the following bootstrapping technique to select the significantly high CDMIs for further investigation (Vergara et al., 2017). We first computed the state probabilities and joint probabilities across all data (subject × window) from the clustering results on each DFDC. We used these values to computer one CDMI for each DFDC pair. Note that the CDMI of a DFDC with itself was ignored, as it is merely the entropy for that DFDC. We bootstrapped these CDMI values using 10^9^ iterations. The significant CDMI threshold was chosen at 5% level. The DFDC pairs with CDMI values higher than this threshold were selected for further analysis. The CDMIs also had right skewed distribution and were transformed similar to the state probabilities and entropy, before being fit to a linear model to examine group difference.

We also assessed the relationship of the SZ’s DFDC entropy and the significant CDMIs with both the patient PANSS scores and CPZ dosage equivalents. We correlated the CPZ dosage equivalents (in log-scale) with each DFDC entropy and significant CDMI. The CPZ dosage equivalents were log-transformed because their original distribution had very low “peakedness” or kurtosis. We also correlated the patient PANSS scores (positive, negative and general separately) with each DFDC entropy and the significant CDMI.

## Results

**FIGURE 2:**
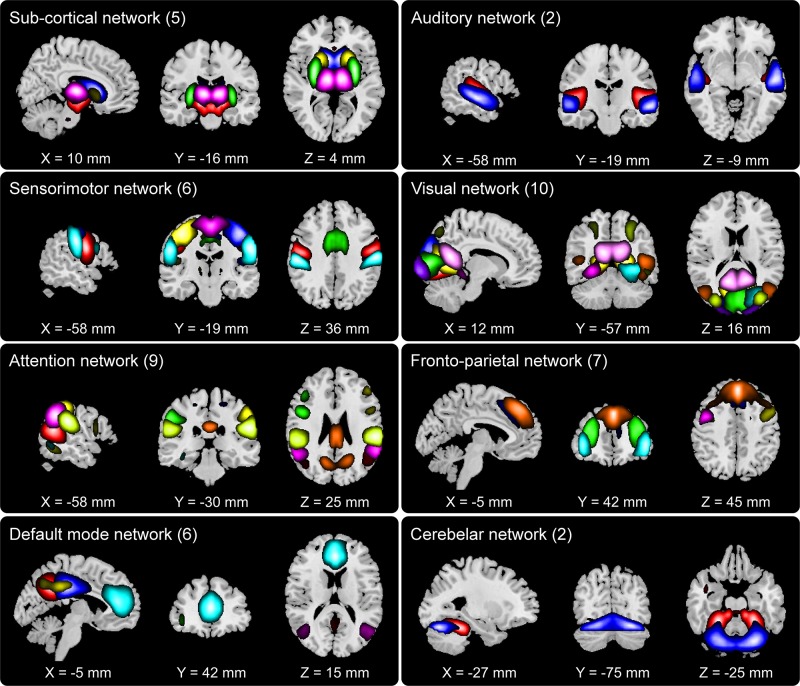
Composite view of the 47 spatially independent group-level functional networks categorized into eight functional domains: subcortical (SC, 5 networks), auditory (AUD, 2), sensorimotor (SM, 6), visual (VIS, 10), attention (ATTN, 9), frontoparietal (FRN, 7), default mode (DMN, 6) and cerebellar (CB, 2). Intensity of the color represents *z*-scores. Functional network labels and peak activation coordinates can be found in prior work (Damaraju et al., 2014).

We set out to examine group differences between HCs and SZs using the information theoretic framework of brain connectivity. The first step in this process was to estimate the brain networks using group ICA. Figure 2 shows the 47 networks grouped into eight functional domains, i.e., SC (5 networks), AUD (2), SM (6), VIS (10), ATTN (9), FRN (7), DMN (6), and CB (2) domains. Out of these, AUD and CB, having two ICs each, were excluded from further analysis. More details such as the anatomical labels and peak activation coordinate of these networks can be found in prior work (Damaraju et al., 2014) and in the Supplementary Table 1.

### Group Difference in DFDC State Probabilities

**FIGURE 3:**
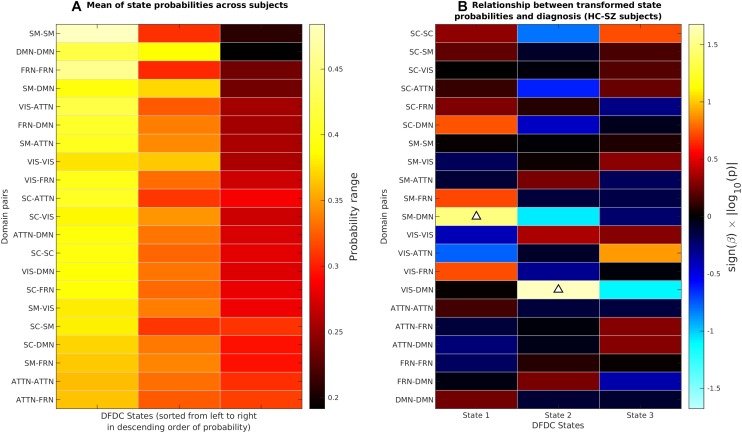
**(A)** The DFDC state probabilities averaged across all subjects for each of the *K* = 3 states and DFDC domain pairs obtained using *K*-means clustering. The domain pairs are sorted from top to bottom in the order of increasing entropy. **(B)** Results obtained by regressing the diagnosis labels (HC-SZ) of the subjects on each of the DFDC state probabilities. The color intensity indicates *sign*(β)×|*log*⁡10(*p*)| where β is the coefficient of regression and *p* is the *p*-value corresponding to the *t*-statistic of the coefficient. Upward arrows indicate that in two cases SZ have significantly higher state probabilities than HCs (*p* < 0.05, uncorrected).

We determined the DFDC states via *K*-means clustering and examined the group differences in the subject state probabilities. The six domains under experiment generated $\left(\begin{array}{l} 6\\ 2 \end{array}\right)=21$ domain pairs. Supplementary Figure 1 shows the three cluster centroids or states for each of the 21 DFDC. Figure 3A shows the mean probabilities for the three states across all subjects. The DFDC are sorted from top to bottom in the order of increasing entropy, and the mean probabilities in each row are sorted from left to right in decreasing order. We observed that the state probabilities had a right-skewed distribution (see Supplementary Figures 2, 4, 6) and transformed them to standard normal distributions (see Supplementary Figures 3, 5, 7). Figure 3B shows the result of linear regression on the transformed state probabilities with diagnosis (HC-SZ) as the independent categorical variable. The color intensity indicates *sign*(β)×|*log*⁡10(*p*)| where β is the coefficient of regression and *p* is the *p*-value corresponding to the t-statistic of the coefficient. Results indicate that SZs have significantly higher (transformed) state 1 probabilities in the SM-DMN DFDC and (transformed) state 2 probabilities in VIS-DMN DFDC at *p <* 0.05 level (uncorrected).

### Group Difference in DFDC and dFNC Entropy

**FIGURE 4:**
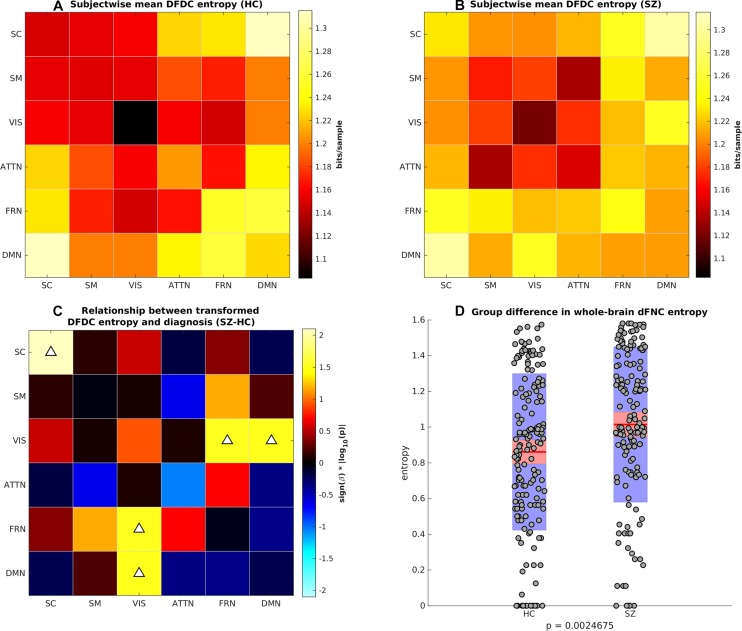
**(A)** Subject-wise mean entropy for HCs, **(B)** subject-wise mean entropy for SZs, **(C)** group difference results from regressing diagnosis on subject-wise entropy. The color intensity indicates *sign*(β)×|*log*⁡10(*p*)| where β is the coefficient of regression and *p* is the *p*-value corresponding to the *t*-statistic of the coefficient. Upward arrows on the positive quantities indicate SZs have significantly higher entropy than HCs (*p* < 0.05, uncorrected). **(D)** Boxplots showing whole-brain dFNC entropy in HC vs. SZ. SZs have significantly higher dFNC entropy than HCs.

Next, we computed the DFDC entropies from the state probabilities using Eq. 1 and examined the group differences in the entropy of the 21 DFDCs. We observed that the entropy had left-skewed distribution (see Supplementary Figure 8) and transformed them to standard normal distributions (see Supplementary Figure 9). Figures 4A,B show the mean entropy across all HCs and SZs for different DFDC. Figure 4C shows the result of linear regression on the transformed entropy with diagnosis (HC-SZ) as the independent categorical variable. The color intensity indicates *sign*(β)×|*log*⁡10(*p*)| where β is the coefficient of regression and *p* is the *p*-value corresponding to the *t*-statistic of the coefficient. Results indicate that SZs have significantly higher entropy than HCs in the SC-SC, FRN-VIS and DMN-VIS DFDC at *p* < 0.05 level (uncorrected). Figure 4D shows the entropy for HCs and SZs computed from the whole-brain dFNC. We observed that the dFNC entropy is also significantly higher in SZs (*p* = 0.0025), commensurate with the majority of the DFDC entropy.

### Group Difference in Cross-Domain Mutual Information

**FIGURE 5:**
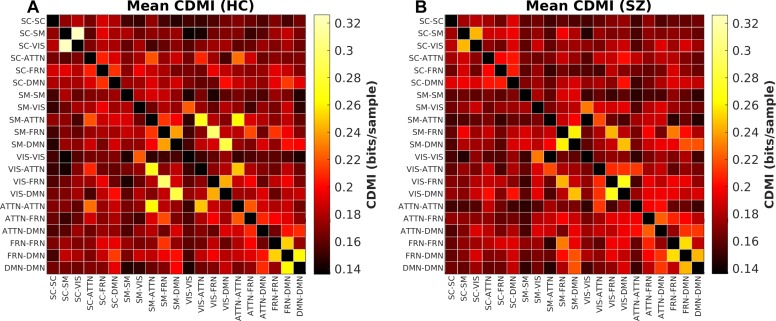
**(A)** Mean CDMI between different DFDC for HCs, **(B)** for SZs.

**FIGURE 6:**
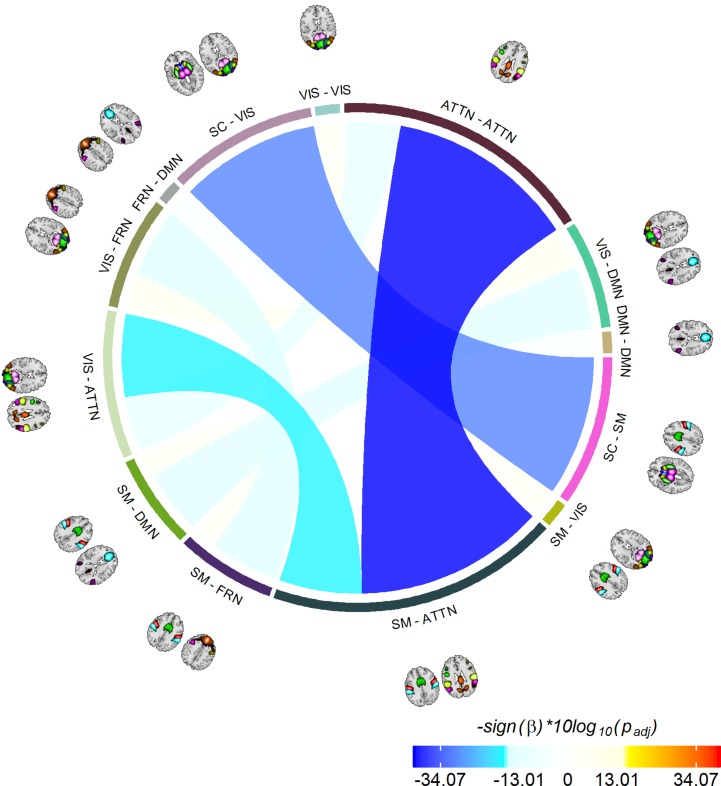
Significant group differences between HCs and SZs. Showing 10 CDMI between 13 different pairs of DFDC which had significantly high CDMI value (determined via bootstrapping). The color of the links indicates *sign*(β) and the width indicates *sign*(β)×|*log*⁡10(*p*)|, where β is the regression slope and *p* is the *p*-value corresponding to the diagnosis variable corrected using FDR correction for multiple comparisons. Negative β values indicate decreased CDMI in SZs, with the three darkest/widest links showing the pairs of DFDC which show significant group difference in CDMI at *p* < 0.05 level.

The last information theoretic measure we examined was the CDMI between each pair of DFDC. We used the state probabilities in Eq. 3 to determine the CDMI between two DFDC and examined the group differences. Pairwise CDMI between 21 DFDC under experiment resulted in $\left(\begin{array}{l} 21\\ 2 \end{array}\right)=210$ CDMI values. Figure 5 show the mean CDMI across HCs and SZs. We observed that when measured across all subjects, the CDMI values were below or equal to 0.2871 (see Supplementary Figure 10). Using bootstrapping, we chose the minimum significant CDMI threshold of 0.099612 at 5% level and performed statistical analysis on the 10 DFDC pairs whose CDMI were higher than this threshold. More details about these 10 CDMI are provided in Supplementary Table 2. We observed that the CDMI showed a right-skewed distribution (see Supplementary Figure 11) and hence transformed them to standard normal distributions (see Supplementary Figure 12). Figure 6 shows a chord diagram of the results of linear regression on the transformed CDMI values with subject diagnosis (HC-SZ) as the independent categorical variable. The color intensity of the connecting links between a pair of DFDC indicates *sign*(β) and the width of the links indicates *sign*(β)×|*log*⁡10(*p*)| where β is the coefficient of regression and *p* is the *p*-value corresponding to the *t*-statistic of the coefficient. The *p*-values were controlled for FDR when conducting multiple comparisons. Results indicate that in 7 out of 10 DFDC pairs under consideration, SZs have lower CDMI than HCs. Three of those are statistically significant at *p* < 0.05 level after FDR correction, which are SC-SM vs. SC-VIS, SM-ATTN vs. VIS-ATTN and SM-ATTN vs. ATTN-ATTN DFDC pairs. This enables us to draw the conclusion that SZs demonstrate significantly lower CDMI, and higher independence, than HCs between these DFDC states.

### Correlation With Medication and Symptoms

We found that the CPZ dosage equivalent of the patients was not significantly correlated with any of the DFDC entropy or the significant CDMIs of the patients. The linear correlation coefficient between the two and the FDR-corrected *p*-values for testing the hypothesis of no correlation are presented in Supplementary Figures 14, 15. We found that the PANSS positive scores of the patients were significantly correlated with the VIS-FRN vs. VIS-DMN CDMI (*R* = 0.27, *p* = 0.0084, FDR corrected). The linear correlation coefficients and FDR corrected *p*-values are added in Supplementary Figures 16, 17. We did not find any significant correlation between the PANSS positive scores and any DFDC entropy, or PANSS general and negative scores of the patients and any of the DFDC entropy or the significant CDMIs after FDR correction for multiple comparisons.

## Discussion

We set out to utilize the information theoretic framework of DFDC (Vergara et al., 2017) to examine how the information processing between the brain functional domains are impacted in schizophrenia. We specifically looked at the transformed entropy in the subject-wise time-varying DFDC patterns, and the CDMI between each pair of those DFDC patterns. We found that SZs show significantly higher entropy than HCs within multiple DFDC. It suggests that there is higher uncertainty or randomness of the DFDC patterns in the diseased brain. Furthermore, SZs demonstrate significantly lower CDMI than HCs between several DFDC pairs. It presents a compelling evidence of higher statistical independence between the activity of different DFDC in the diseased brain.

There are numerous studies hypothesizing aberrant brain function in schizophrenia (Aleman and Kahn, 2005; Brunet-Gouet and Decety, 2006; Juckel et al., 2006; Bassett et al., 2008; Murray et al., 2008; Sommer et al., 2008; Wolf et al., 2009; Esslinger et al., 2013; Fusar-Poli et al., 2013; van den Heuvel et al., 2013; Kahn et al., 2015). Our work is one of the first to explore information sharing at the functional domain level in schizophrenia, and our findings corroborate the notion of the aberrant nature of functional connectivity. We also examined entropy in whole-brain dFNC which has not been reported in the literature. We found that SZs have significantly higher entropy than HCs at both whole-brain and functional domain level.

Evidence of weaker CDMI in SZs implies that connectivity among different pairs of functional domains, or DFDC, have less influence on each other’s domain activation patterns in SZs. Note that we selected a significant CDMI threshold via bootstrapping and ran linear regression on the selected CDMI, because the CDMI for most of the DFDC pairs are very low (indicating higher independence). It can be argued that the results passed the test for multiple comparisons only because of the lower number of comparisons. Hence, we also examined the group difference in every CDMI and corrected for multiple comparisons, instead of selecting a few based on a bootstrapped threshold. We still found that SZs have significantly lower CDMI than HCs in the SM-ATTN vs. ATTN-ATTN CDMI, while showing a general trend toward lower CDMI in SZs compared to HCs (Supplementary Figure 13). Miller et al. (2016a) has previously explored information processing among functional domains in schizophrenia and found that auditory-visual-sensorimotor network in the healthy population showed increased sensitivity to connectivity in the other functional domains. The authors derived two novel metrics (distributional dissimilarity and specificity) based on the transition probabilities of the inter-domain and intra-domain correlation patterns which can capture information flow among the domains. Their major finding – that these metrics are reduced in SZs – is in accordance with our findings. However, we have come to this conclusion by employing information theoretic concepts which are well-defined and broadly used in other fields. Moreover, previous authors used metrics based on transition probability of the connectivity states, whereas we have employed the entropy of the whole sequence of the connectivity states.

We ran some additional tests to investigate the site effect on CDMI and entropy. Specifically, we extended our statistical models to include both diagnosis (2 levels) and site (7 levels) factors, then performed an N-way analysis of covariance using Matlab “anovan” function, followed by a multiple comparison of the stats using the “multcompare” function. We have added the results in Supplementary Figure 18 which shows the 2 cases where we found significant differences between the marginal CDMI means with the site variable considered. In our original manuscript, we identified both these CDMIs to be significantly different between HC and SZ with the site effect not considered. From similar analysis on entropy, site difference was observed in the SC-VIS entropy. Given these results, we do not see significant influence of site on the results in the current setting.

It bears discussion what our findings imply for SZ and brain disorders. From previous literature we know of hyper connectivity abnormalities in the SC region (Damaraju et al., 2014; Vergara et al., 2019) and thus it is not a surprise that we see entropy effects in the SC domain. Our results indicate that SC hyper connectivity in SZ translates into higher uncertainty (higher entropy). Given that the SC domain encompass thalamus and putamen, this could point to an increase of information relay in the cortico-thalamic loops. A possible explanation is that the cortical regions in charge of sensory processing are sending an extra amount of signaling to the cortico-thalamic loops in SZ. This might overwork the information relay functions of the striatal and thalamic regions which responds to this demand by enhancing functioning and increasing the amount of information (larger entropy) processed. This overworking of the brain seems to be determined between the SC and VIS regions and two domains (FRN and DMN). Similar impaired VIS-SC-FRN processing has been previously reported in SZ (Sehatpour et al., 2010; John et al., 2018). In our study, we also discover impaired VIS-DMN processing. The patient PANSS positive score shows significant correlation with the VIS-FRN vs. VIS-DMN CDMI. The literature supports this evidence of link between positive symptoms such as visual hallucination and abnormal DMN activity in SZ (Jardri et al., 2013) as well as Parkinson’s disease (Yao et al., 2014; Shine et al., 2015). It is possible that overworking the SC domain enhances important information among VIS, DMN and FRN domains, but reduces information sharing among other domains in order to compensate the workload. Example of this reduction was observed in the SM-SC vs. SC-VIS CDMI which might have diminished workload to be able to dedicate more resources for other areas more active during resting state. By leveraging information theoretic principles, our work provides some novel insights about the uncertainty of the dFNC and DFDC states in SZ.

Next, we discuss some of the weaknesses and limitations in this study. Firstly, we have imposed the number of clusters, *K* = 3 when clustering the DFDC patterns using the *K*-means algorithm. The number *K* was chosen by observing the elbow criterion. We also experimented by varying *K* between {3,4,5}, as well as by including the AUD and CB functional domains in the analysis. The results agree for various settings, i.e., SZs tend to have higher entropy and lower CDMI than HCs. For different settings we found different sets of DFDC to be significant, but some of the DFDCs, such as SC-SC show higher entropy in SZs, and SM-ATTN vs. ATTN-ATTN CDMI tends to be lower in SZs irrespective of different settings. Secondly, the assignment of different networks to the functional domains tends to be subjective and consensus-based among researchers depending on the anatomy and prevalent knowledge about the function of such networks. An important future direction will be to motivate optimal choice of the number of clusters, clustering method, and a replicable method of assignment of functional domains in the information theoretic framework and beyond. Finally, we observed that the effect sizes of the entropy/CDMI group differences are not very large (see Supplementary Figures 9, 12). The effect sizes range between 0.09 and 0.30 for the DFDCs showing significant group differences in entropy, and the linear regression results do not pass correction for multiple comparisons. However, for the CDMI group comparisons, the effect sizes are higher (0.29−0.46) and the linear regression results indeed pass FDR correction for multiple comparisons to corroborate our conclusions.

Since its introduction 70 years ago, information theory has been adopted and appropriated in many different scientific domains, including neuroscience where the study of neural information flow sees major application of the information theoretic principles. In recent years the interest in dynamic nature of the human brain and its implication in brain disorders has peaked. Brain disorders such as schizophrenia as well as aging and dementia are strongly indicative of the deterioration of storage and transmission mechanism of information in the human brain. In this work, we have generated novel insights about the uncertainty of the dFNC and DFDC states in schizophrenia by leveraging information theoretic principles. Further work in this area may help us decipher what language distant brain regions use to “talk” to each other, as well as how this interaction is impaired in brain diseases (Vergara and Calhoun, 2017). The study of the complex yet orderly, dynamic function of the healthy human brain and the lack thereof in mental illness can hopefully benefit from the application of the simple, yet elegant information theoretic framework such as one introduced in our work.

## Data Availability

The datasets for this manuscript are not publicly available. We plan to make all of the connectivity matrices, results, and code available to the public. While we would also like to make the raw data available, it is not our study and unfortunately there are still IRB restrictions which prevent such sharing for now. Requests to access the datasets should be directed to https://www.nitrc.org/projects/fbirn.

## Ethics Statement

The data used in this study were collected under the Functional Biomedical Informatics Research Network (FBIRN) phase-III study at the following sites: University of California, Irvine (UCI), University of California, Los Angeles (UCLA), The University of New Mexico (NM), The University of Iowa (IA), University of Minnesota (MN), Duke University/University of North Carolina, University of California, San Diego (UCSD; healthy subjects only), and the University of California, San Francisco (UCSF). Informed consent was obtained from the participants according to the guidelines set by the Internal Review Boards at each site.

## Author Contributions

MS, VV, and VC designed the project. MS wrote the manuscript. ED performed the preprocessing, group ICA, and dFNC analyses. MS, VV, and VC performed the DFDC and statistical analyses, interpreted the results, and edited the manuscript.

## Conflict of Interest Statement

The authors declare that the research was conducted in the absence of any commercial or financial relationships that could be construed as a potential conflict of interest.

## Abbreviations

ATTN: attention
BOLD: blood oxygenation level dependent
CDMI: cross-domain mutual information
DFDC: dynamic functional domain connectivity
dFNC: dynamic functional network connectivity
DMN: default mode network
EPI: echo planar imaging
FBIRN: functional biomedical informatics research network
FDR: false discovery rate
fMRI: functional magnetic resonance imaging
FNC: functional network connectivity
FRN: frontoparietal
FWHM: full width at half maximum
GIFT: group ICA of fMRI
HCs: healthy controls
ICA: independent component analysis
ICs: independent components
MI: mutual information
MNI: montreal neurological institute
PCA: principal component analysis
PCs: principal components
RMS: root mean square
RSN: resting-state networks
SC: subcortical
SFNR: signal-to-fluctuation noise ratio
SM: sensorimotor
STR: spatio-temporal regression
SZs: schizophrenia patients
TCs: time courses
VIS: visual.
