# Supplementary material for: Decreased Cross-Domain Mutual Information in Schizophrenia From Dynamic Connectivity States
Source: Front Neurosci. 2019 Aug 22;13:873. doi: 10.3389/fnins.2019.00873 (PMC6714616; doi:10.3389/fnins.2019.00873)
Supplement: Supplementary file 1 [file Data_Sheet_1.PDF]

Supplementary Material

1 Supplementary Figures and Tables

1.1 Supplementary Figures

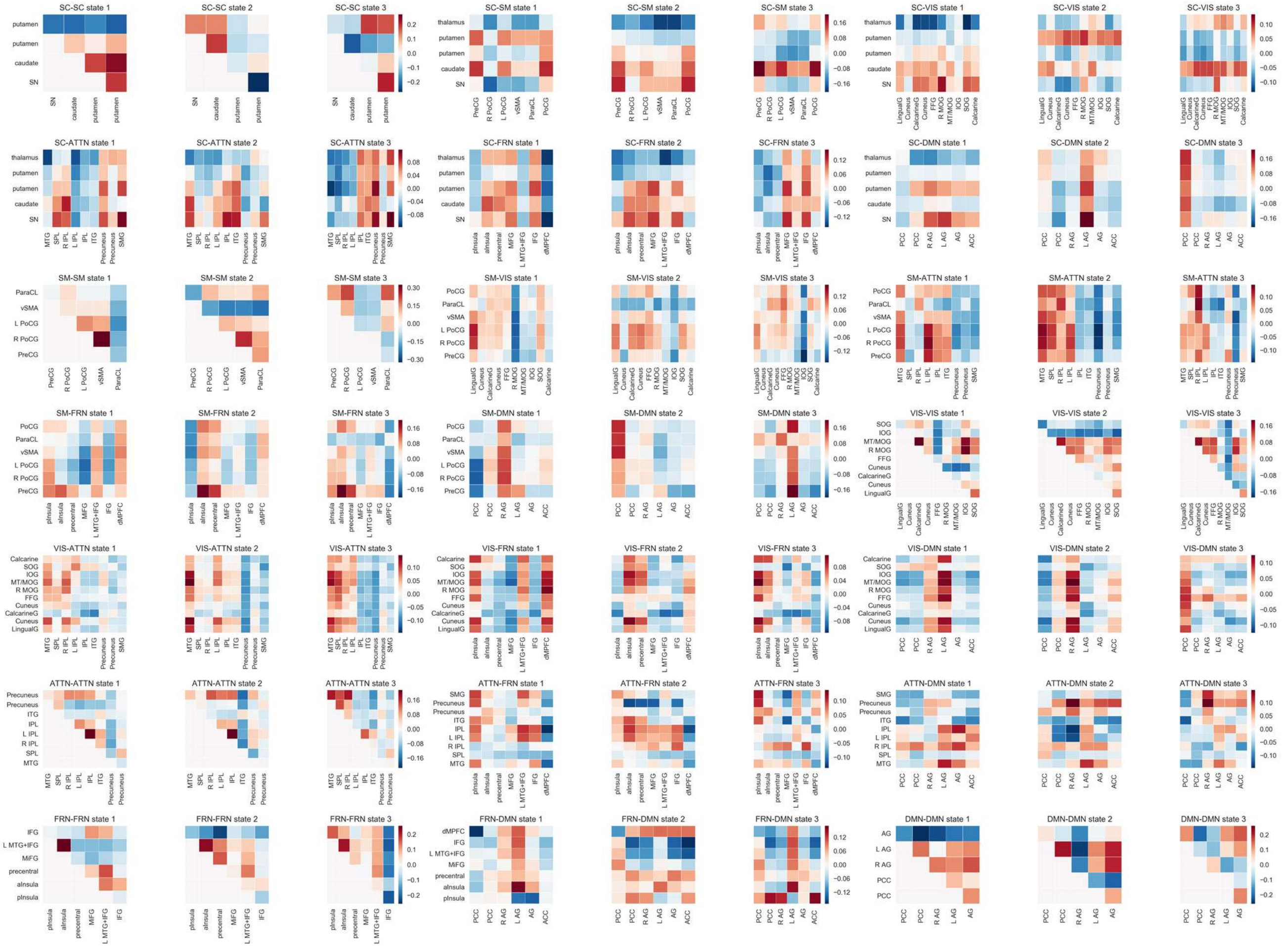

Supplementary Fig. 1. The DFDC centroids or states obtained from K-means clustering on every DFDC across all subjects.

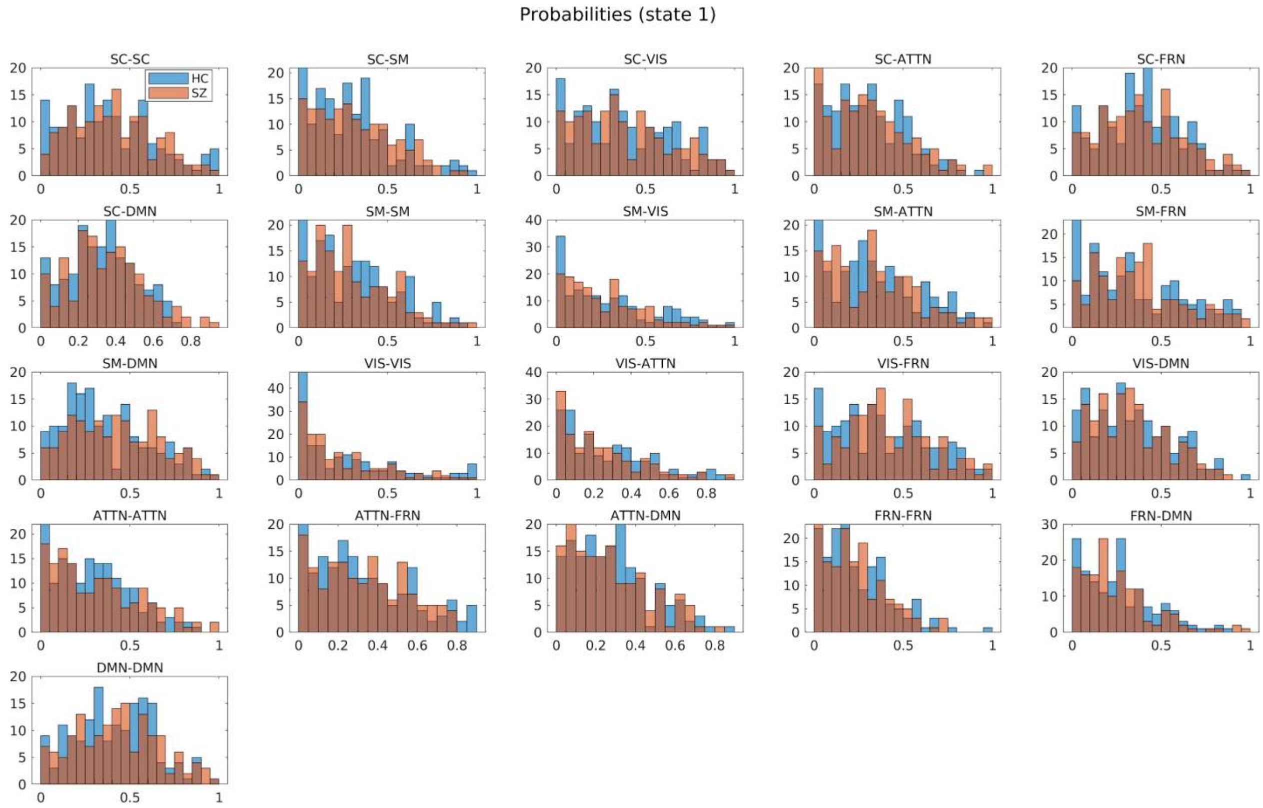

Supplementary Fig. 2. State 1 probability histograms for HC and SZ for different DFDC.

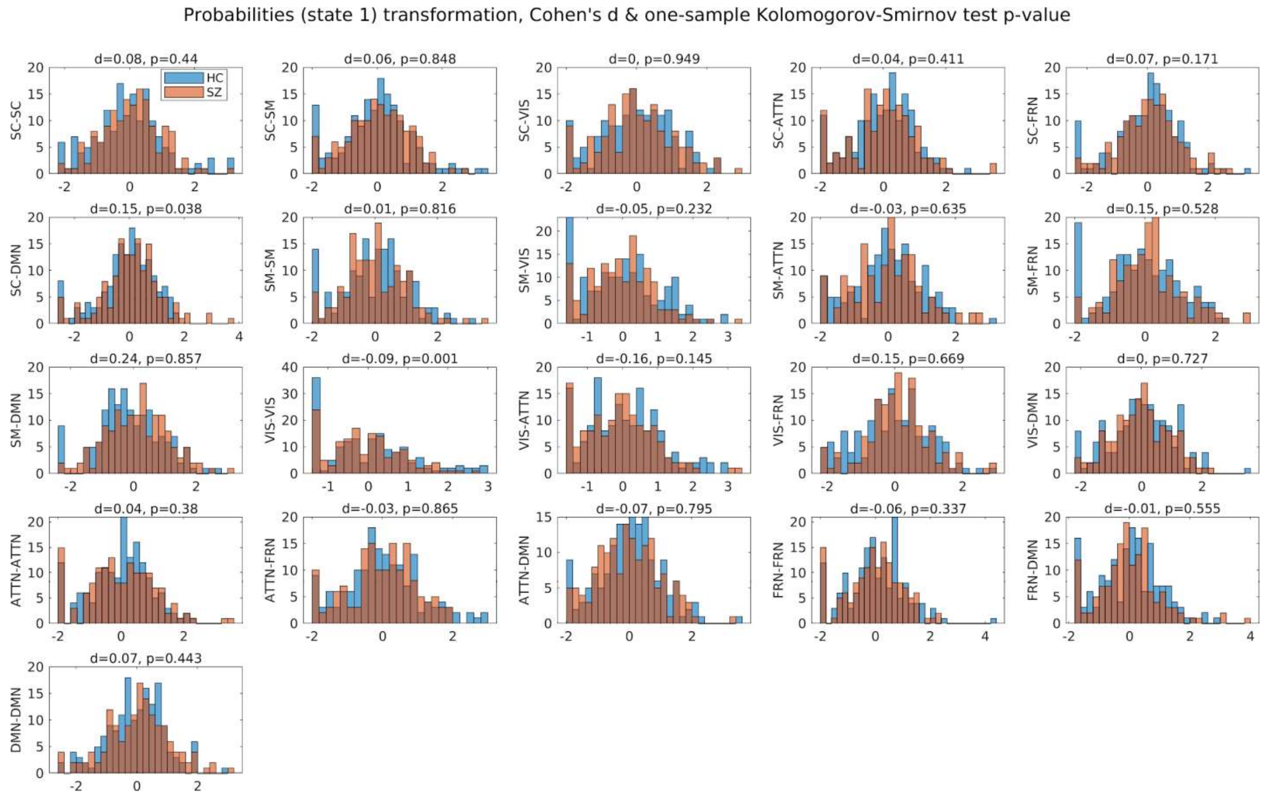

Supplementary Fig. 3. State 1 transformed probability histograms for HC and SZ for different DFDC domain pairs (shown in the y-label). The transformation is $\mathrm{zscore}\left(\mathrm{atanh}\left(\mathrm{rescale}(\mathrm{probability}, -(1-\mathrm{K}), 1-\mathrm{K})\right)\right), \mathrm{K} = 0.1$. The title of each plot shows the Cohen's d measure of effect size (between HC and SZ), and the one-sample Kolomogorov-Smirnov test p-value indicating normality (across all subjects). $\mathrm{p} > 0.05$ indicates that the data come from a standard normal distribution.

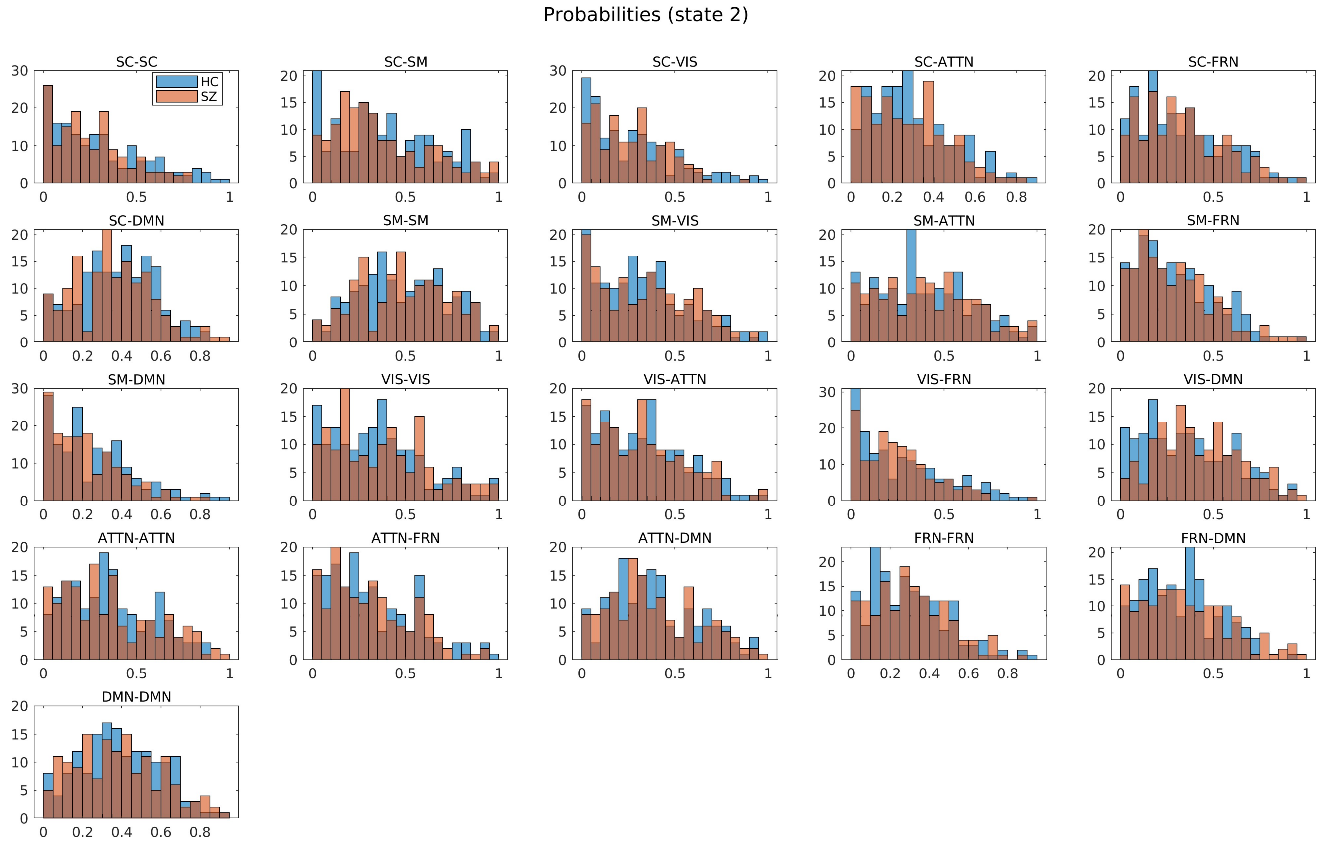

Supplementary Fig. 4. State 2 probability histograms for HC and SZ for different DFDC.

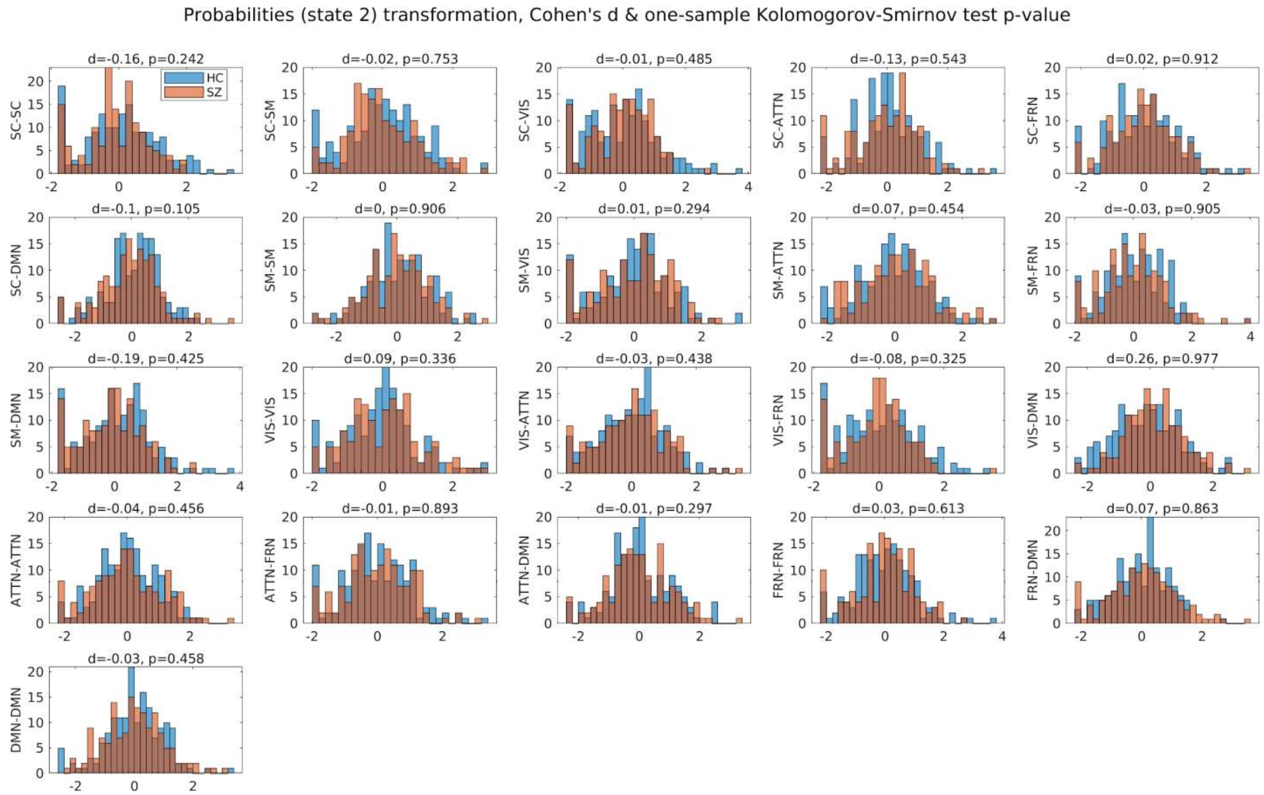

Supplementary Fig. 5. State 2 transformed probability histograms for HC and SZ for different DFDC domain pairs (shown in the y-label). The transformation is $\text{zscore}\left(\text{atanh}\left(\text{rescale}(\text{probability}, -(1-\text{K}), 1-\text{K})\right)\right), \text{K} = 0.1$. The title of each plot shows the Cohen's d measure of effect size (between HC and SZ), and the one-sample Kolomogorov-Smirnov test p-value indicating normality (across all subjects). $\text{p} > 0.05$ indicates that the data come from a standard normal distribution.

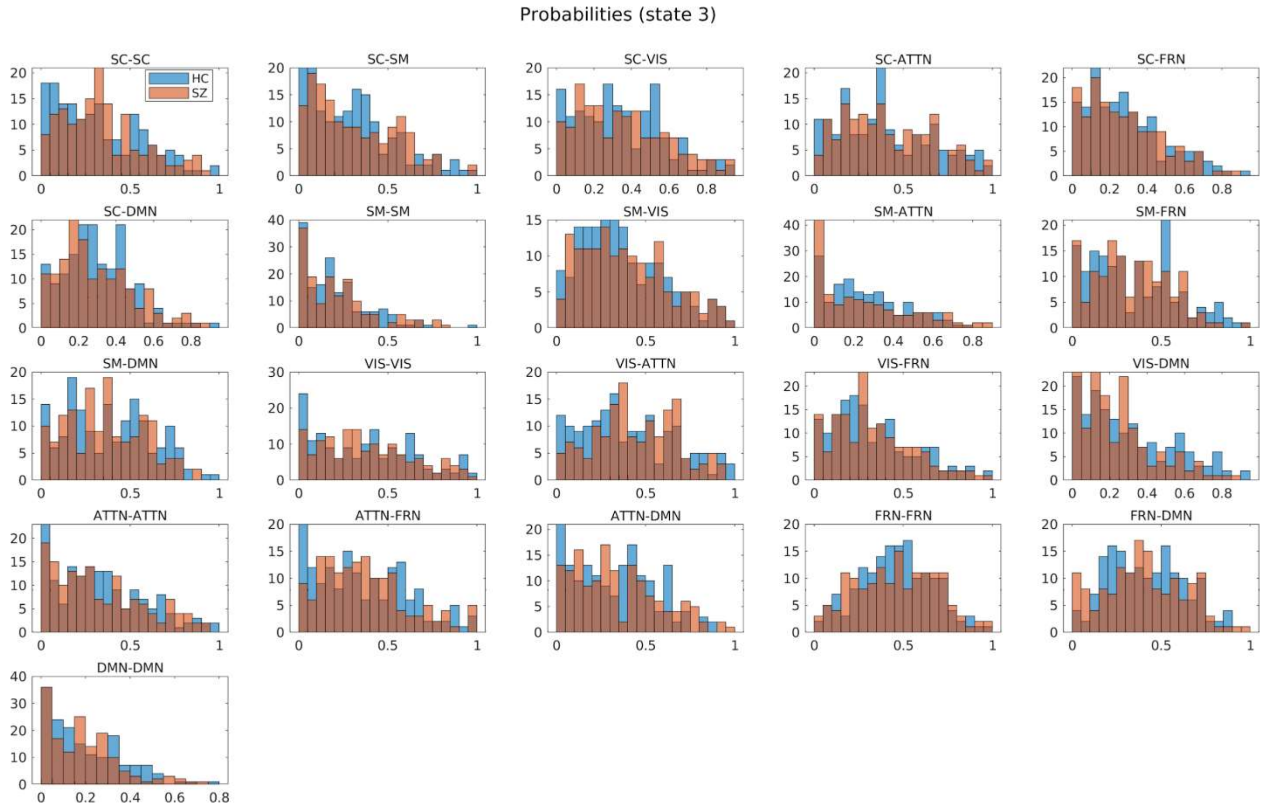

Supplementary Fig. 6. State 3 probability histograms for HC and SZ for different DFDC.

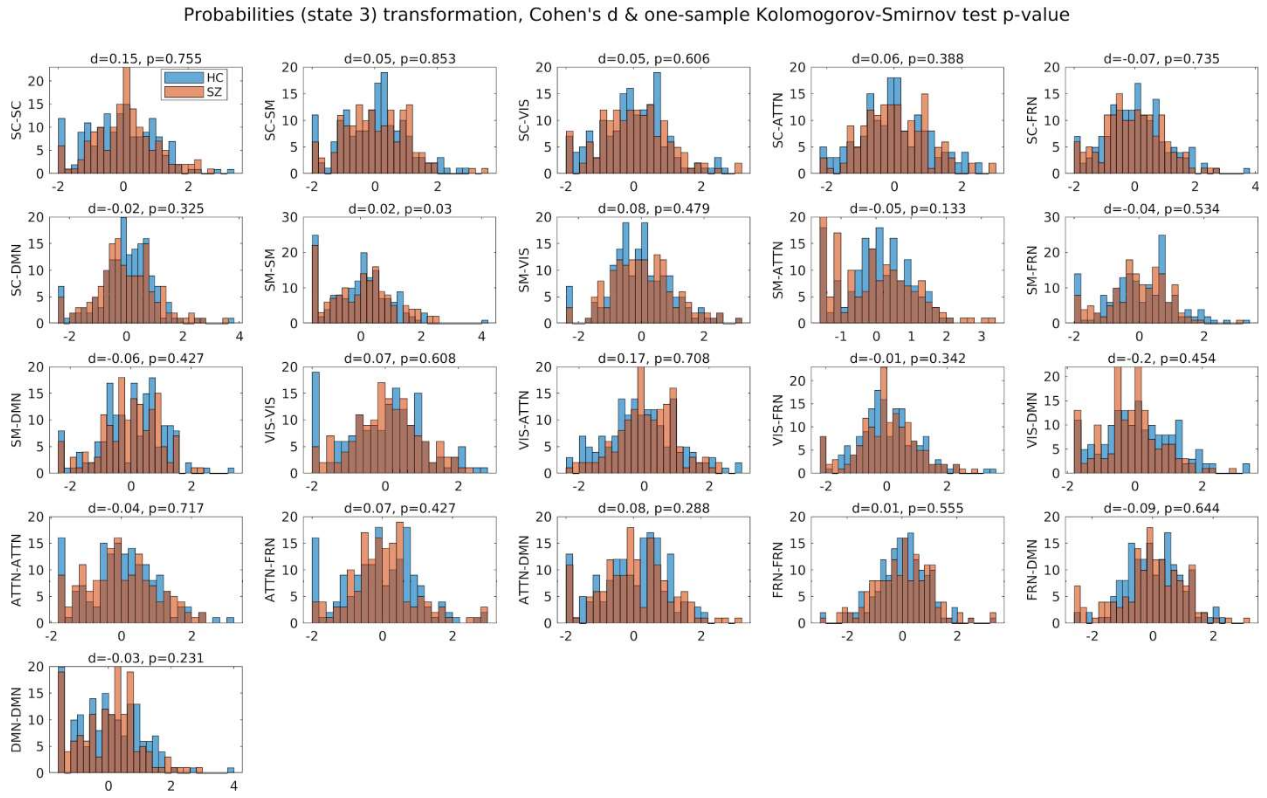

Supplementary Fig. 7. State 3 transformed probability histograms for HC and SZ for different DFDC domain pairs (shown in the y-label). The transformation is $\mathrm{zscore}\left(\mathrm{atanh}\left(\mathrm{rescale}(\mathrm{probability}, -(1-\mathrm{K}), 1-\mathrm{K})\right)\right), \mathrm{K}=0.1$. The title of each plot shows the Cohen's d measure of effect size (between HC and SZ), and the one-sample Kolomogorov-Smirnov test p-value indicating normality (across all subjects). $\mathrm{p} > 0.05$ indicates that the data come from a standard normal distribution.

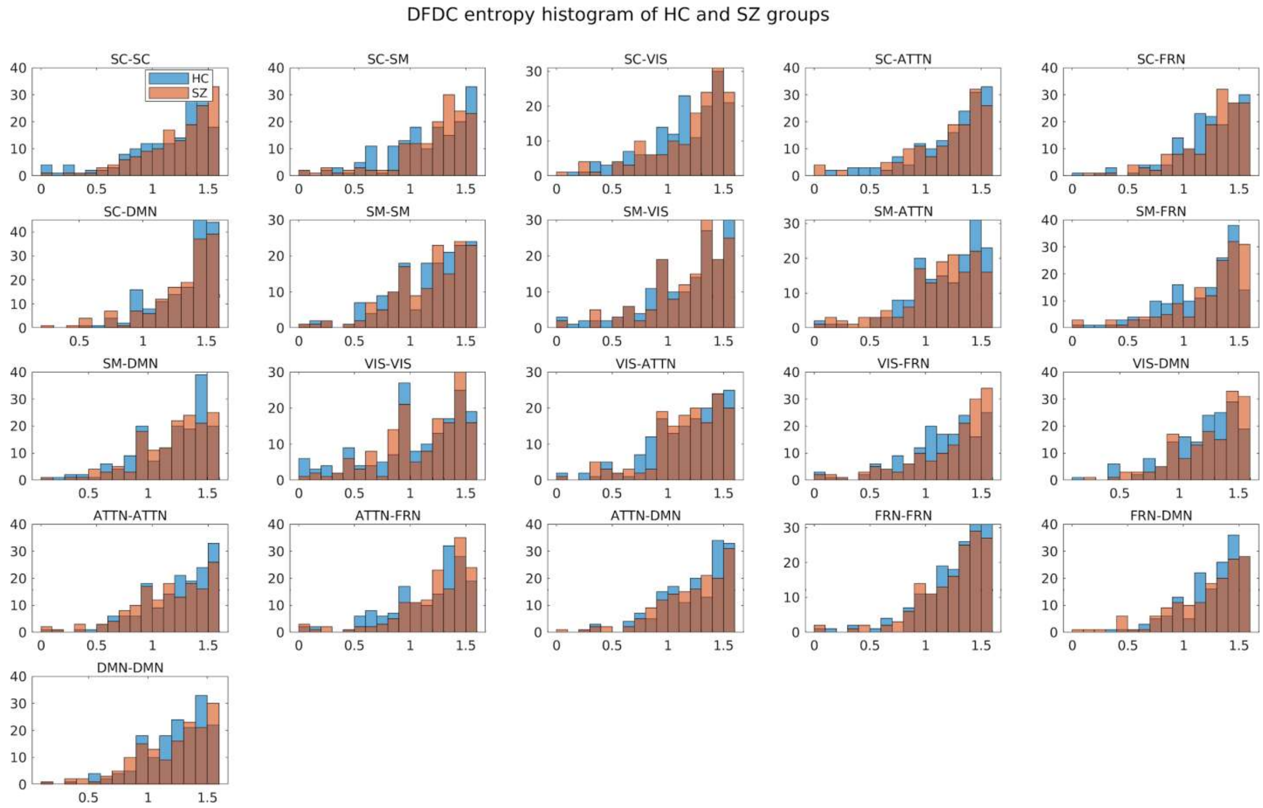

Supplementary Fig. 8. Entropy histograms for HC and SZ for different DFDC.

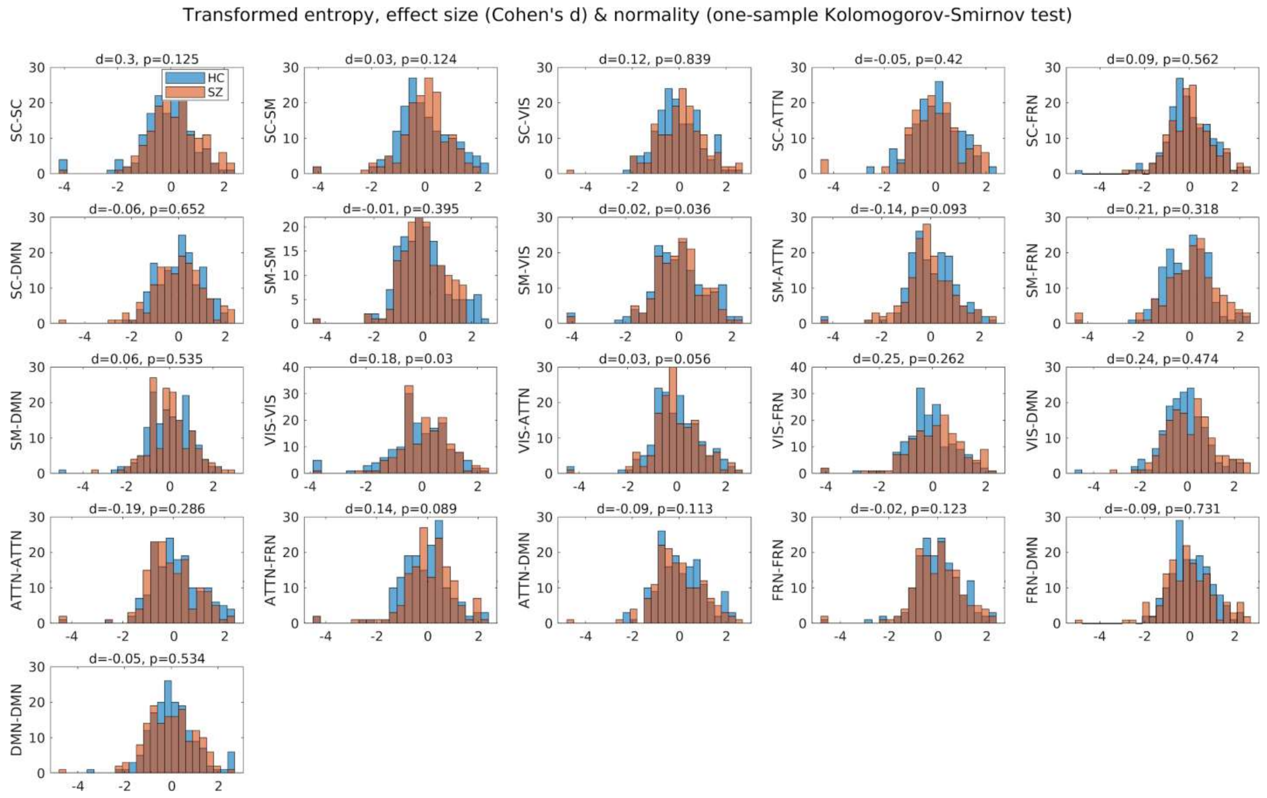

Supplementary Fig. 9. Transformed entropy histograms for HC and SZ for different DFDC domain pairs (shown in the y-label). The transformation is $\mathrm{zscore}\left(\mathrm{atanh}\left(\mathrm{rescale}(\mathrm{entropy}, -(1-\mathrm{K}), 1-\mathrm{K})\right)\right), \mathrm{K} = 0.015$. The title of each plot shows the Cohen's d measure of effect size (between HC and SZ), and the one-sample Kolomogorov-Smirnov test p-value indicating normality (across all subjects). $\mathrm{p} > 0.05$ indicates that the data come from a standard normal distribution.

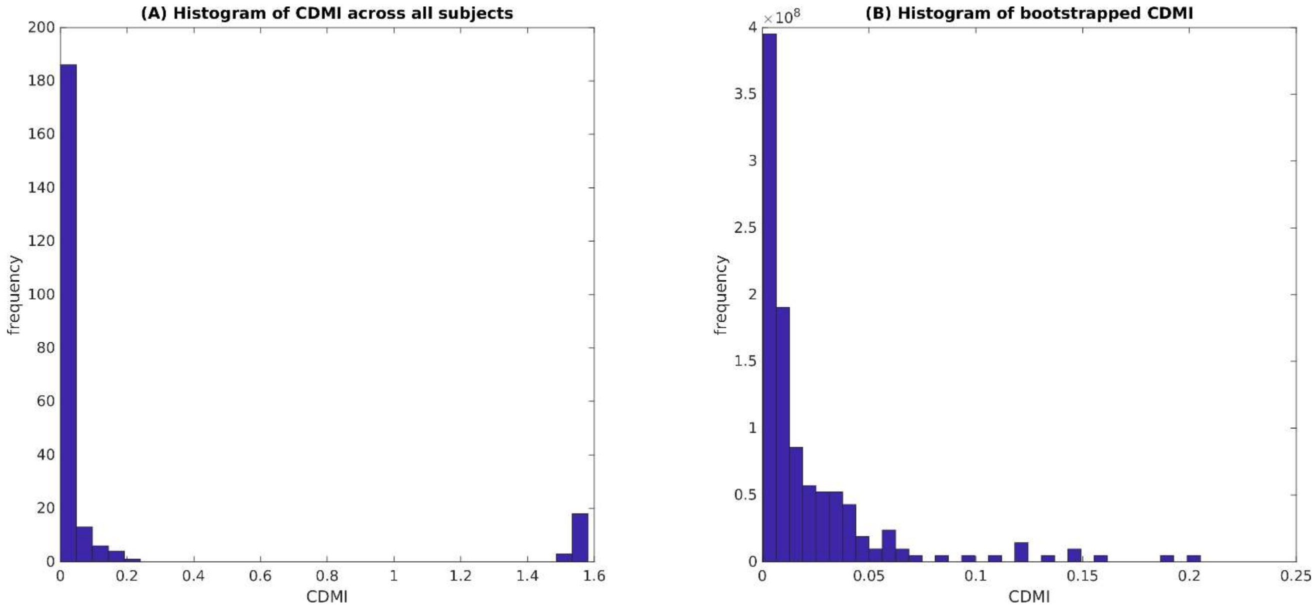

Supplementary Fig. 10. (A) Histogram of CDMI across all subjects shows that only a handful of DFDC pairs are highly independent. These are the CDMI between a DFDC with itself, hence corresponding to the entropy of the associated domain pair. (B) For the rest of the (smaller) CDMI, the 5% significant threshold was determined at $I>0.099612$ by bootstrapping. Figure shows the histogram of those bootstrapped CDMI values.

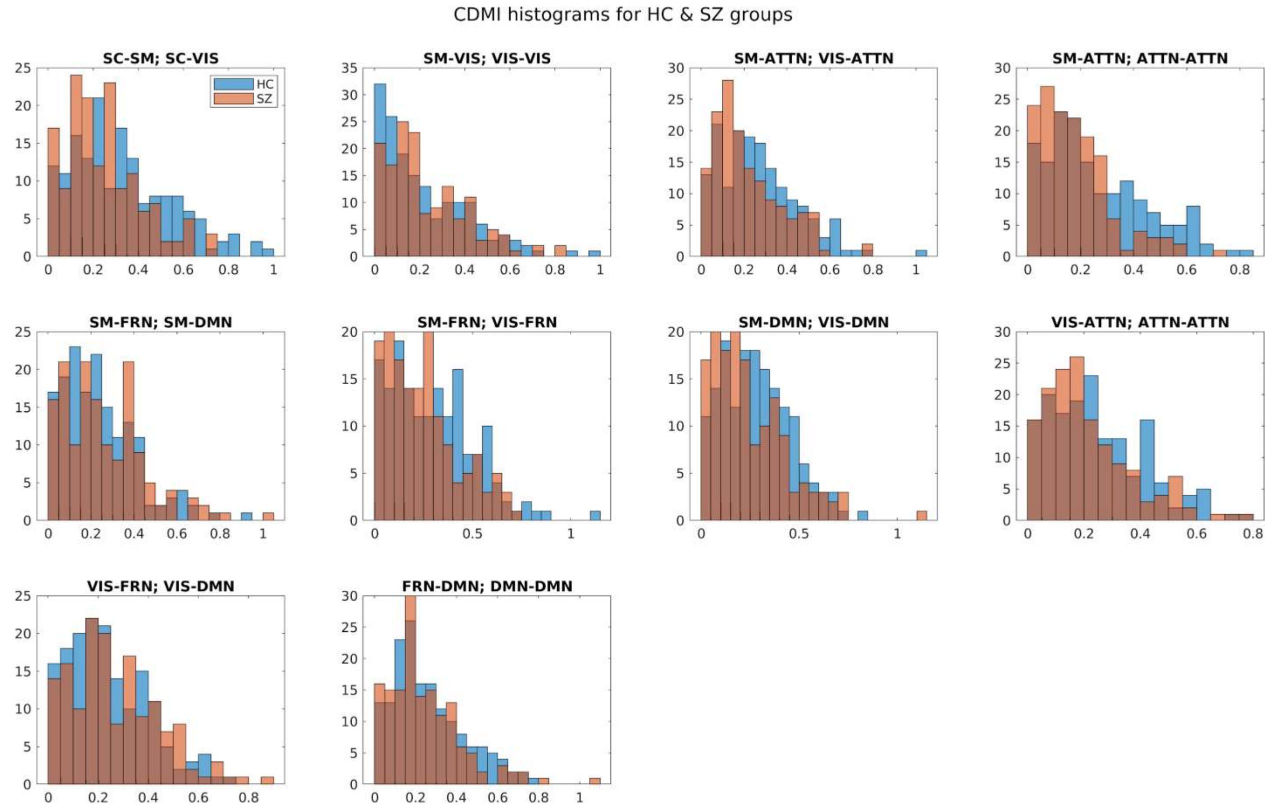

Supplementary Fig. 11. CDMI histograms for HC and SZ for between 10 DFDC pairs. These 10 CDMI have significantly high values, greater than the threshold determined by bootstrapping.

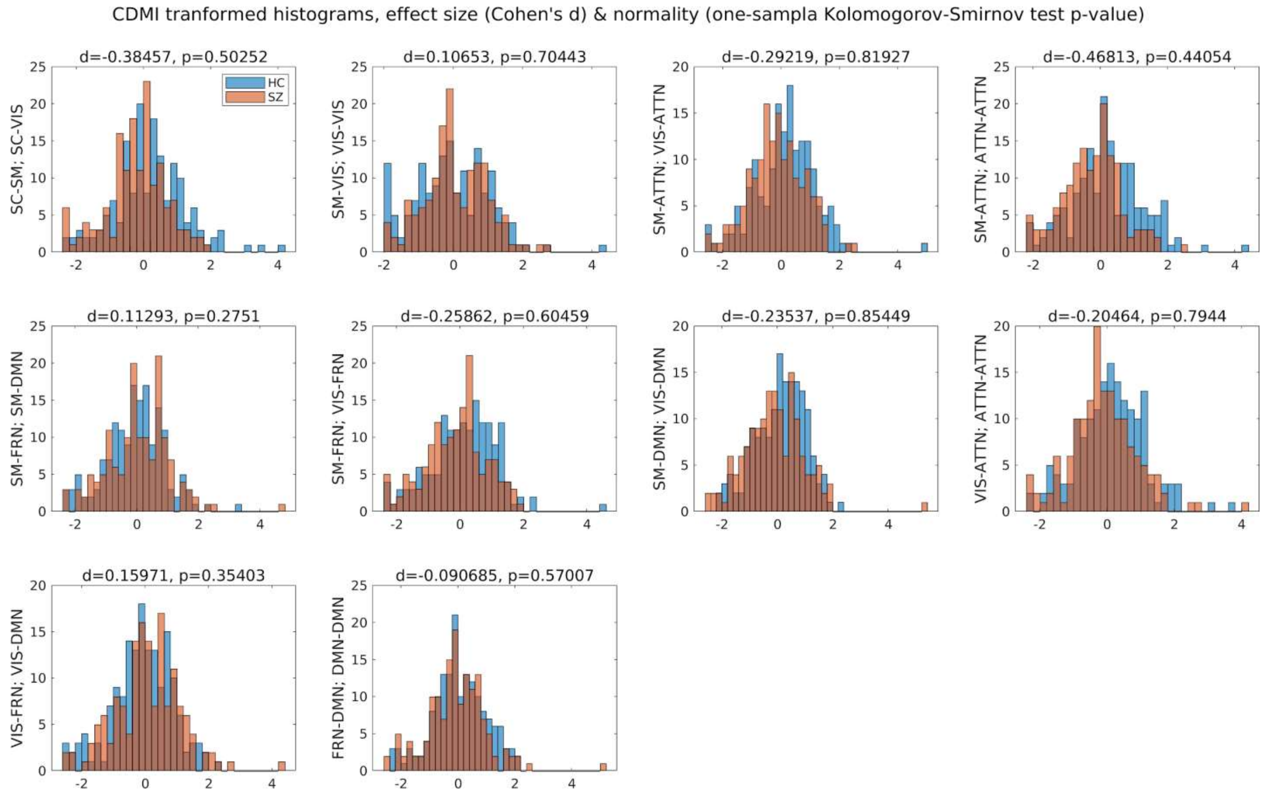

Supplementary Fig. 12. Transformed CDMI histograms for HC and SZ for 10 DFDC pairs (shown in the y-label). The transformation is $\text{zscore}\left(\text{atanh}\left(\text{rescale}(\text{CDMI}, -(1-\text{K}), 1-\text{K})\right)\right), \text{K} = 0.05$. The title of each plot shows the Cohen's d measure of effect size (between HC and SZ), and the one-sample Kolomogorov-Smirnov test p-value indicating normality (across all subjects). $p => 0.05$ indicates that the data come from a standard normal distribution.

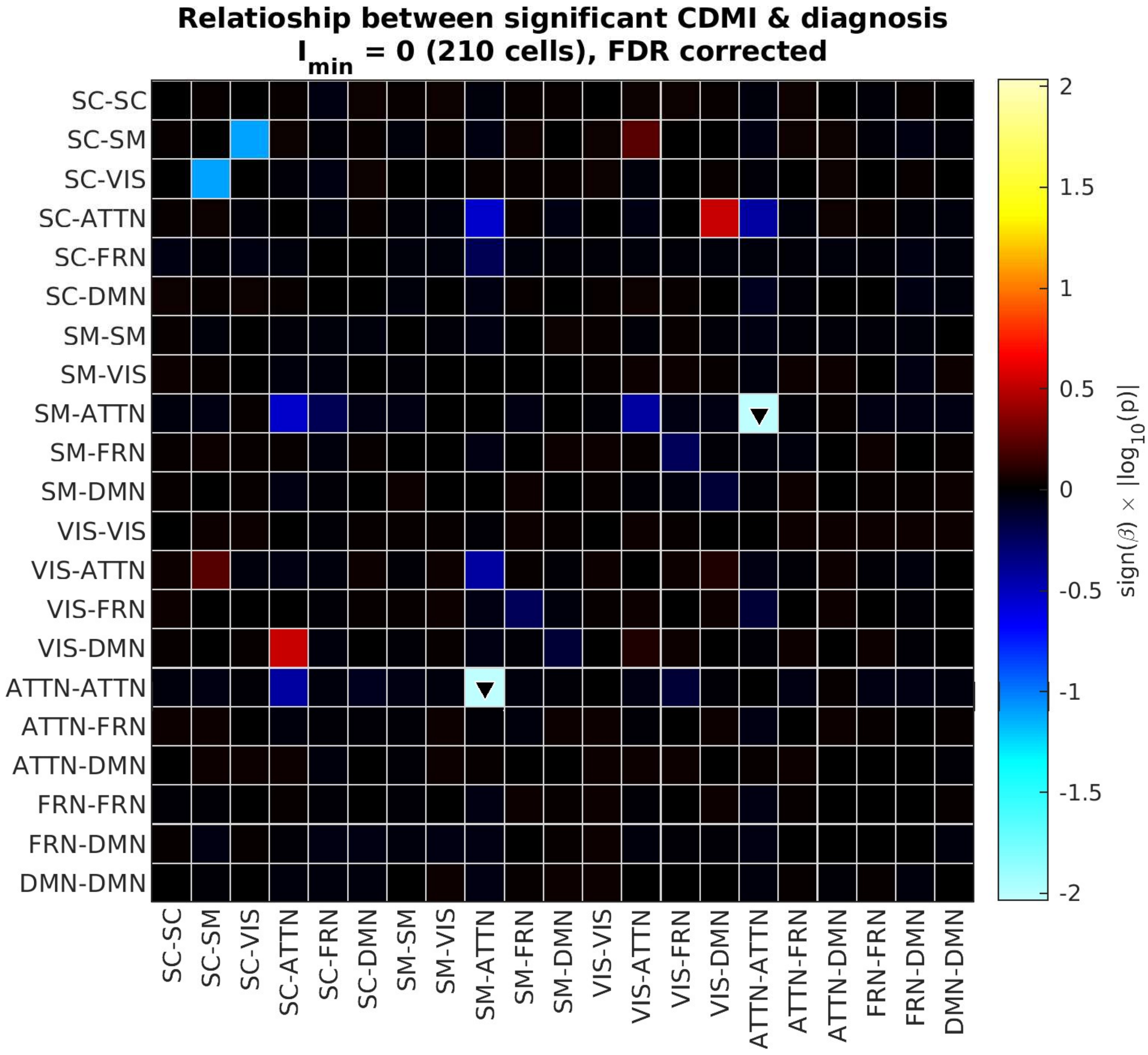

Supplementary Fig. 13. Significant group differences in CDMI between HCs and SZs. The color of the cells indicatessign$(\beta) \times |\log 10(p)|$, where β is the regression slope and p is the p-value corresponding to the diagnosis, corrected using FDR correction for multiple comparisons. After taking all the CDMI into consideration (instead of thresholding) and correcting for multiple comparisons, SM-ATTN vs ATTN-ATTN CDMI shows significant group difference.

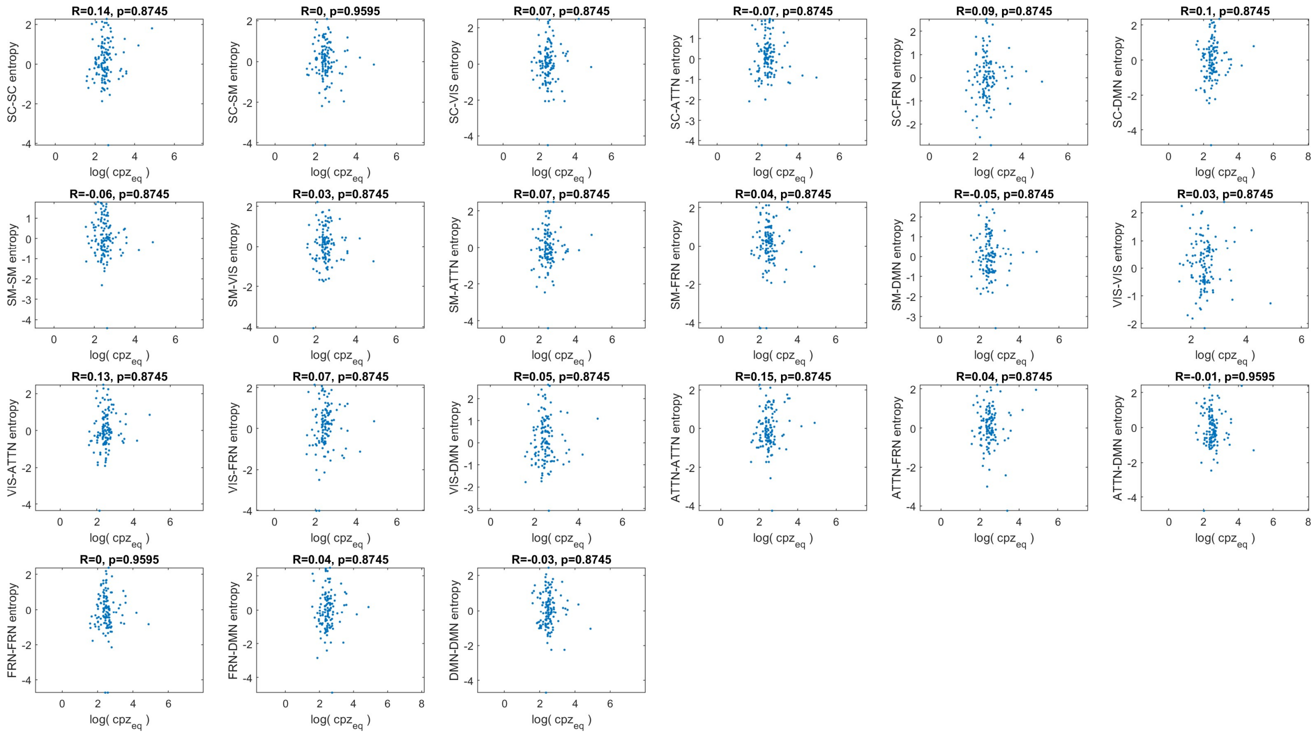

Supplementary Fig. 14. Correlation between the log of chlorpromazine (CPZ) dosage equivalents for the patients and (transformed) DFDC entropy. R is the linear correlation coefficient between the two and p indicates the FDR corrected p-values for testing the hypothesis of no correlation against the alternative hypothesis of a nonzero correlation.

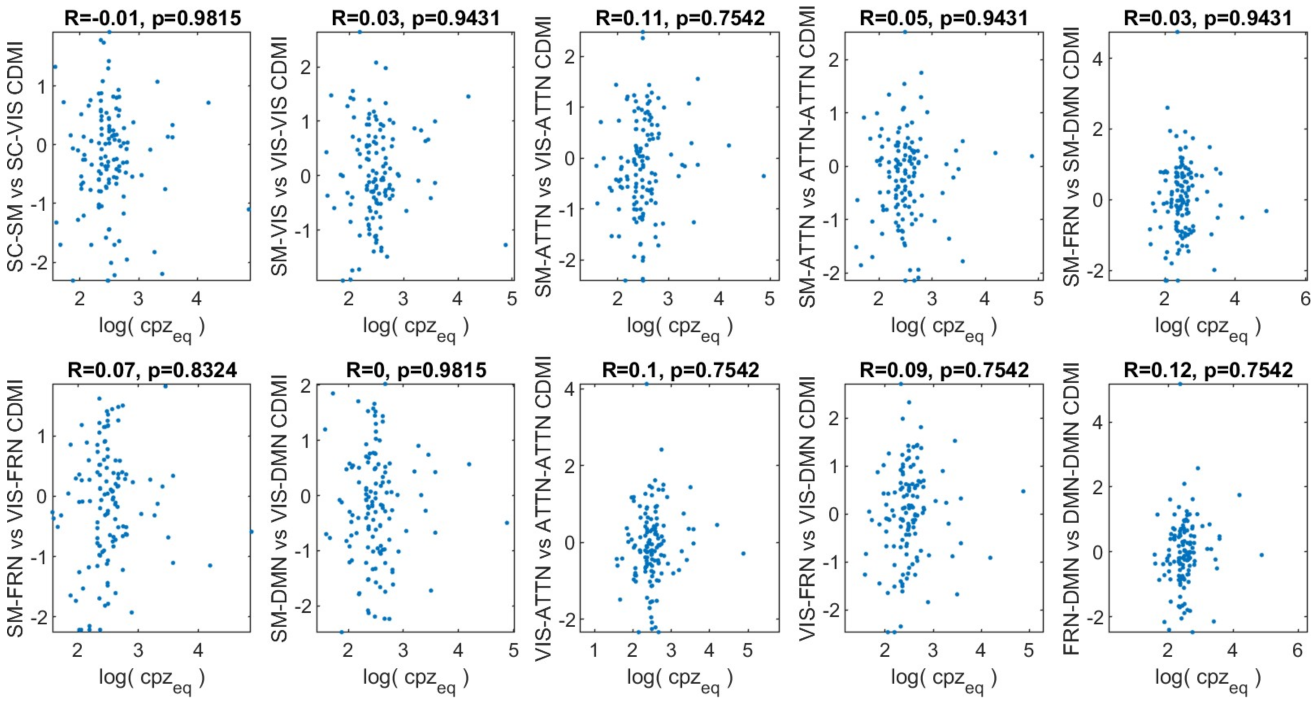

Supplementary Fig. 15. Correlation between the log of chlorpromazine (CPZ) dosage equivalents for the patients and significant (transformed) CDMIs. r is the linear correlation coefficient between the two and p indicates the FDR corrected p-values for testing the hypothesis of no correlation against the alternative hypothesis of a nonzero correlation.

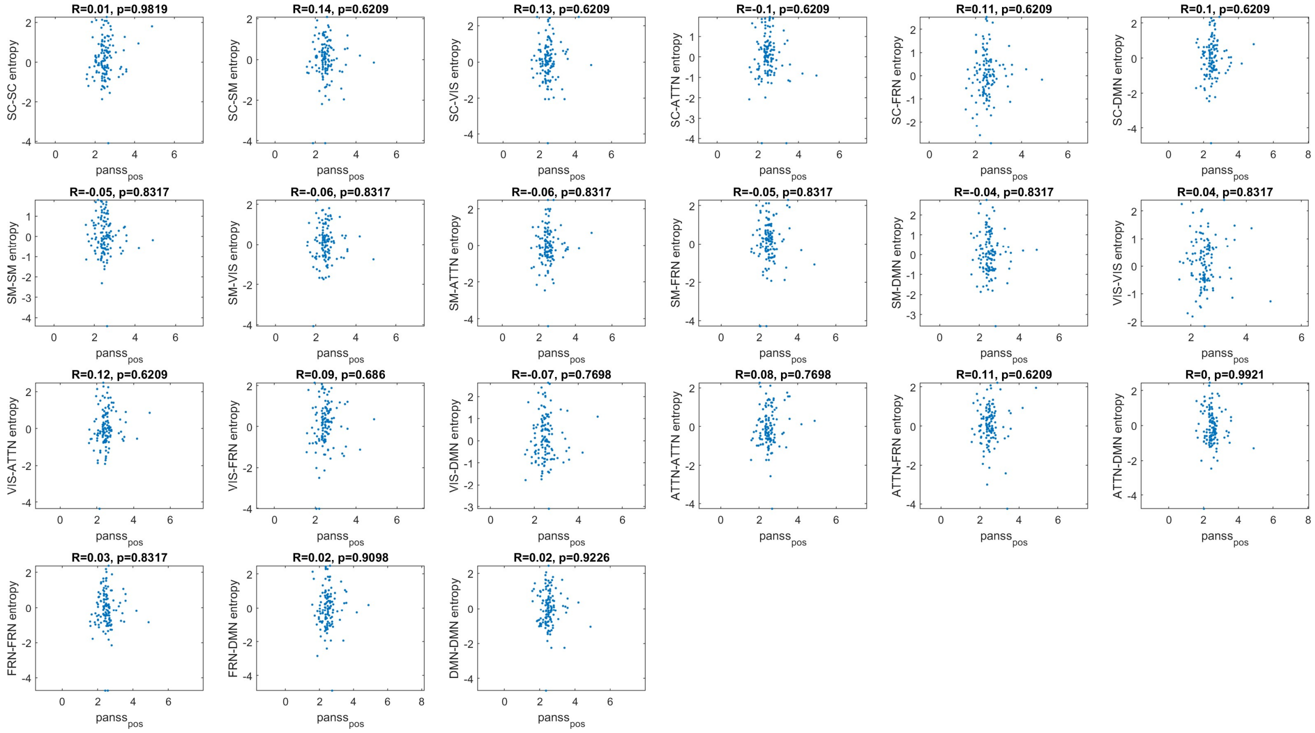

Supplementary Fig. 16. Correlation between the modified Positive Symptom Scale (PANSS positive) score for the patients and (transformed) DFDC entropy. R is the linear correlation coefficient between the two and p indicates the FDR corrected p-values for testing the hypothesis of no correlation against the alternative hypothesis of a nonzero correlation.

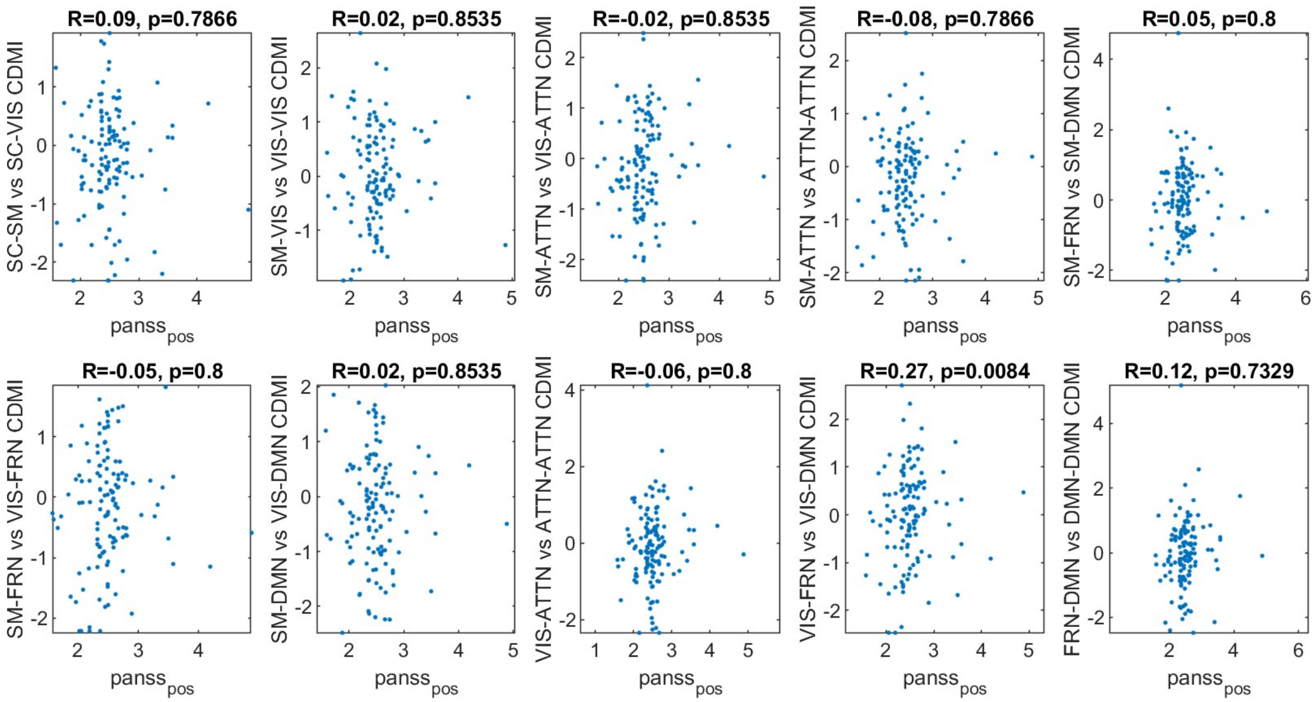

Supplementary Fig. 17. Correlation between the modified Positive Symptom Scale (PANSS positive) score for the patients and significant (transformed) CDMIs. R is the linear correlation coefficient between the two and p indicates the FDR corrected p-values for testing the hypothesis of no correlation against the alternative hypothesis of a nonzero correlation.

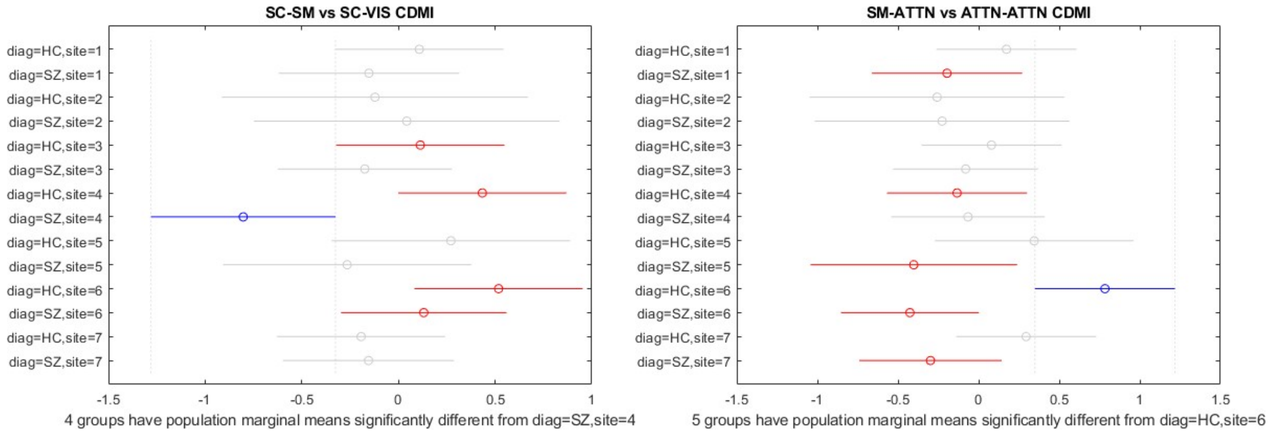

Supplementary Fig. 18. Site effect on the CDMI between SC-SM vs SC-VIS and SM-ATTN vs ATTN-ATTN DFDC pairs. Marginal means are obtained by using “multcompare” Matlab function, following N-way analysis of covariance using the model CDMI~diagnosis+site.

1.2 Supplementary Tables

Supplementary Table 1. Full form name and functional domain of the networks used throughout the text

Network#	Short name	Full form name	Functional domain
75	SN	Substantia nigra	SC (subcortical)
13	Caudate	Caudate	SC
1	Putamen	Putamen	SC
2	Putamen	Putamen	SC
18	Thalamus	Thalamus	SC
58	STG	Superior temporal gyrus	AUD (auditory)
51	STG	Superior temporal gyrus	AUD
5	PreCG	Precentral gyrus	SM (sensorimotor)
6	R PoCG	Right postcentral gyrus	SM
10	L PoCG	Left postcentral gyrus	SM
74	vSMA	Supplementary motor area	SM
9	ParaCL	Paracentral lobule	SM
59	PoCG	Postcentral gyrus	SM
76	LingualG	Lingual gyrus	VIS (visual)
78	Cuneus	Cuneus	VIS
43	CalcarineG	Calcarine	VIS
7	Cuneus	Cuneus	VIS
57	FFG	Fusiform gyrus	VIS
91	R MOG	Right mid. occipital gyrus	VIS
42	MT/MOG	Mid. temporal/mid. occipital gyrus	VIS
20	IOG	Inferior occipital gyrus	VIS
60	SOG	Superior occipital gyrus	VIS
12	Calcarine	Calcerine	VIS
80	MTG	Mid. temporal gyrus	ATTN (attention)
24	SPL	Superior parietal lobule	ATTN
66	R IPL	Right inferior parietal lobule	ATTN
94	L IPL	Left inferior parietal lobule	ATTN
89	IPL	inferior parietal lobule	ATTN
63	ITG	Inferior temporal gyrus	ATTN
35	Precuneus	Precuneus	ATTN
40	Precuneus	Precuneus	ATTN
96	SMG	Supramarginal gyrus	ATTN
41	pInsula	Posterior insula	FRN (frontal)
28	aInsula	Anterior insula	FRN
34	precentral	Precentral	FRN

21	MiFG	Mid. inferior frontal gyrus	FRN
61	L MTG+IFG	Left mid. temporal gyrus + inferior frontal gyrus	FRN
65	IFG	Inferior frontal gyrus	FRN
69	dMPFC	Dorsomedial prefrontal cortex	FRN
30	PCC	Posterior cingulate cortex	DMN (default mode network)
47	PCC	Posterior cingulate cortex	DMN
84	R AG	Right angular gyrus	DMN
95	L AG	Right angular gyrus	DMN
90	AG	Angular gyrus	DMN
53	ACC	Anterior cingulate cortex	DMN
46	cereb	Cerebellum	CB (cerebellar)
88	cereb	Cerebellum	CB

Supplementary Table 2. Functional domain pairs with significantly high CDMI

**Domain Pair 1**	**Domain Pair 2**	**Entropy 1**	**Entropy 2**	**CDMI**	**Regression result**		
					**beta**	**p-value**	**FDR corrected p-value**
SC-SM	SC-VIS	1.57771	1.569898	0.205165	-0.38423	0.000613	0.003067
SM-VIS	VIS-VIS	1.574837	1.565477	0.147524	0.106644	0.345894	0.384327
SM-ATTN	VIS-ATTN	1.561564	1.554392	0.156784	-0.29081	0.009814	0.032714
SM-ATTN	ATTN-ATTN	1.561564	1.581677	0.121325	-0.45936	3.92E-05	0.000392
SM-FRN	SM-DMN	1.579316	1.55201	0.119982	0.114754	0.3104	0.384327
SM-FRN	VIS-FRN	1.579316	1.566798	0.186737	-0.25688	0.022706	0.056766
SM-DMN	VIS-DMN	1.55201	1.570971	0.147432	-0.23816	0.03477	0.069539
VIS-ATTN	ATTN-ATTN	1.554392	1.581677	0.119436	-0.21139	0.061156	0.101927
VIS-FRN	VIS-DMN	1.566798	1.570971	0.135334	0.159457	0.158355	0.226221
FRN-DMN	DMN-DMN	1.558839	1.512058	0.111952	-0.0885	0.434185	0.434185
